# Targeting YAP1 to improve the efficacy of immune checkpoint inhibitors in liver cancer: mechanism and strategy

**DOI:** 10.3389/fimmu.2024.1377722

**Published:** 2024-03-14

**Authors:** Yuting Gao, Yi Gong, Junlan Lu, Huiqin Hao, Xinli Shi

**Affiliations:** ^1^Laboratory of Integrated Medicine Tumor Immunology, Shanxi University of Chinese Medicine, Taiyuan, China; ^2^Chinese Medicine Gene Expression Regulation Laboratory, State Administration of Traditional Chinese Medicine, Shanxi University of Chinese Medicine, Taiyuan, China; ^3^Basic Laboratory of Integrated Traditional Chinese and Western, Shanxi University of Chinese Medicine, Taiyuan, China

**Keywords:** YAP1, liver cancer, immune checkpoint inhibitors, tumor microenvironment, metabolic reprogramming

## Abstract

Liver cancer is the third leading of tumor death, including hepatocellular carcinoma (HCC) and intrahepatic cholangiocarcinoma (ICC). Immune checkpoint inhibitors (ICIs) are yielding much for sufferers to hope for patients, but only some patients with advanced liver tumor respond. Recent research showed that tumor microenvironment (TME) is critical for the effectiveness of ICIs in advanced liver tumor. Meanwhile, metabolic reprogramming of liver tumor leads to immunosuppression in TME. These suggest that regulating the abnormal metabolism of liver tumor cells and firing up TME to turn “cold tumor” into “hot tumor” are potential strategies to improve the therapeutic effect of ICIs in liver tumor. Previous studies have found that YAP1 is a potential target to improve the efficacy of anti-PD-1 in HCC. Here, we review that YAP1 promotes immunosuppression of TME, mainly due to the overstimulation of cytokines in TME by YAP1. Subsequently, we studied the effects of YAP1 on metabolic reprogramming in liver tumor cells, including glycolysis, gluconeogenesis, lipid metabolism, arachidonic acid metabolism, and amino acid metabolism. Lastly, we summarized the existing drugs targeting YAP1 in the treatment of liver tumor, including some medicines from natural sources, which have the potential to improve the efficacy of ICIs in the treatment of liver tumor. This review contributed to the application of targeted YAP1 for combined therapy with ICIs in liver tumor patients.

## 1 Introduction

As the third leading cause of tumor death in the world, liver tumor is the only one of the top five deadliest tumors to have an annual percentage increase in occurrence (1, 2). Primary liver tumor mainly includes two different histological subtypes, hepatocellular carcinoma (HCC) and intrahepatic cholangiocarcinoma (ICC), with etiology and biological heterogeneity in the clinic (3). Often, the incidence of liver tumor is higher in developing countries (4). The main risk factors of liver tumor include hepatitis B virus (HBV) and hepatitis C virus (HCV) infection, non-alcoholic fatty liver disease, metabolic syndrome, and various dietary exposures (5). According to the analysis of global epidemiological changes in liver tumor, with the prevalence of obesity and the increase in alcohol intake, non-alcoholic fatty liver disease, and alcoholic liver disease have replaced HBV infection as the primary cause (6, 7). Due to the late onset of symptoms, a large proportion of patients with HCC are diagnosed at advanced stages, resulting in an inferior prognosis (8, 9). No longer eligible for curative or locoregional therapies, these patients with advanced liver tumor can only choose to seek systemic treatment (10). Oral sorafenib is the first choice for chemotherapy for advanced liver tumor, but fewer than one-third of patients benefit from treatment and develop drug resistance within six months (11). Traditional treatments such as ablation therapies and chemotherapy cannot effectively improve the prognosis of patients with advanced liver tumor (12).

In the past few years, immune checkpoint inhibitors (ICIs) have revolutionized the systematic treatment of liver tumor (13). The Food and Drug Administration (FDA) approved the combination of atezolizumab (anti-programmed death-ligand 1) and bevacizumab (anti-vascular endothelial growth factor) as first-line treatment for advanced liver tumor because it improved overall survival compared to sorafenib (14, 15). Although the treatment of liver tumor has evolved from single-drug targeted therapy to ICI combined targeted therapy, only a small subset of patients can obtain durable clinical benefits. Therefore, it is still a great challenge to improve the therapeutic effect of ICIs for liver tumor (16).

The effectiveness of ICI therapy for liver tumor largely depends on the tumor microenvironment (TME) (17, 18). The occurrence and development of liver tumor are accompanied by chronic inflammation, which leads to the accumulation of immune cells and cytokines in the liver, forming a hypoxia immunosuppressive TME that supports the growth of tumor cells (19). Metabolic reprogramming is an emerging hallmark of liver tumor. Increasing evidence suggests that metabolic changes in TME are essential to ICI resistance in liver tumor (20). Metabolic reprogramming of liver tumor contributes to the maintenance of immunosuppressive TME, resulting in tumor immune escape (21). Liver tumor TME enhances metabolic states such as glycolysis and lipid metabolism and releases metabolites such as lactic acid and arginine, which seriously inhibit immune cells’ differentiation, proliferation, and activation. There is metabolic competition between tumors and immune cells in TME, which limits the access of immune cells to nutrients and leads to TME acidosis, which hinders the function of immune cells (22).

**Figure 1:**
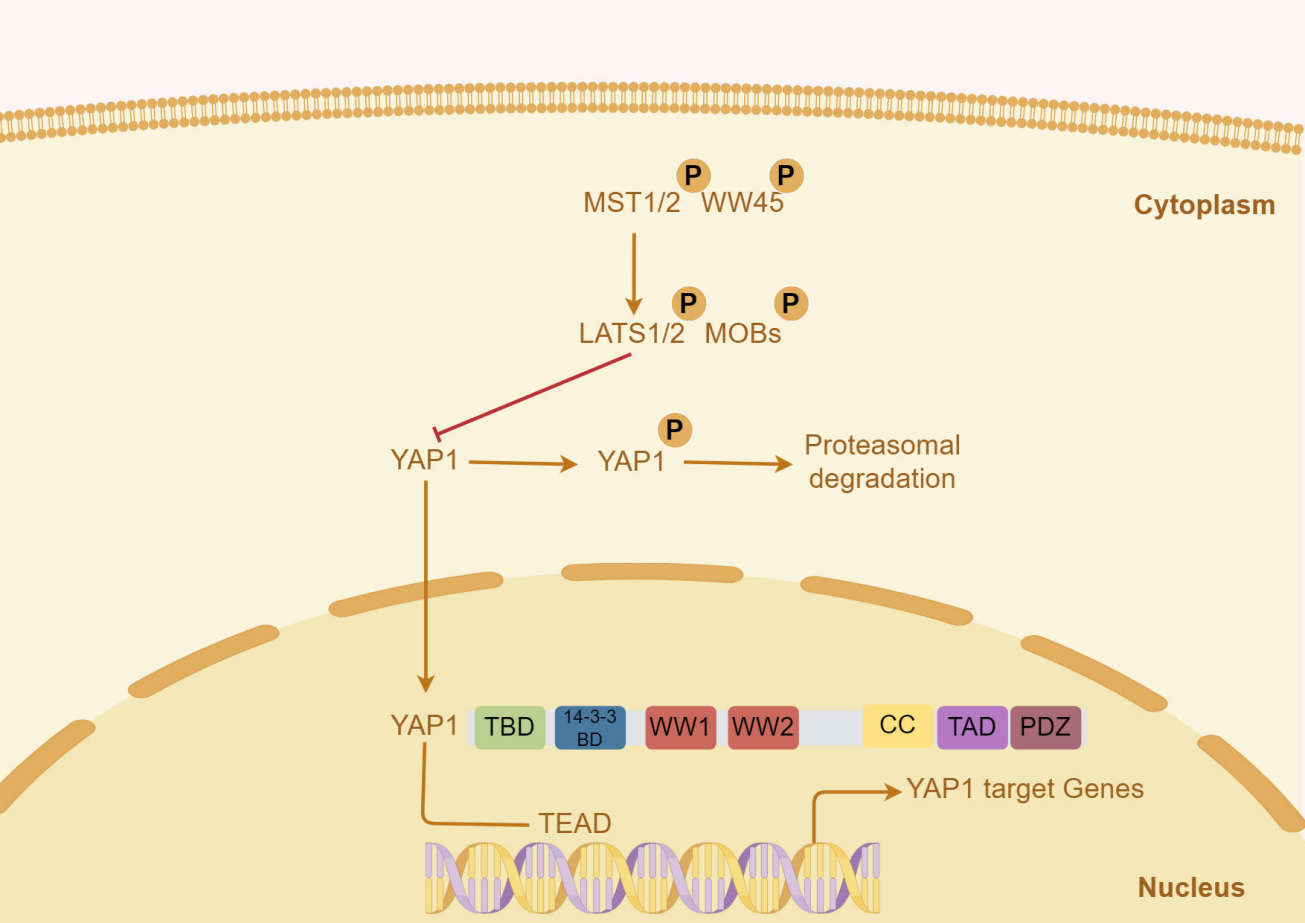
Key signals regulating YAP1 in liver tumor and the structure of human YAP1 protein. In the Hippo pathway, the activated MST1/2 and WW45 complexes phosphorylate, activate the LATS/MOB complex, and then phosphorylate YAP1, resulting in YAP1 translocation and degradation. The structure of the human YAP1 protein mainly includes TBD, TEAD-binding domain; WW, W-containing domain; CC, coiled-coil domain; TAD, transactivation domain.

The Hippo/YAP pathway regulates organ growth and cell proliferation (23). Yes-associated protein (YAP1), a co-transcriptional factor of Hippo/YAP pathway, translocates from the nucleus to the cytoplasm (24). The upstream of YAP1 mainly includes the mammalian STE20-like protein (MST) 1/2, the mammalian ortholog of Salvator (WW45/SAV), the large tumor suppressor homolog (LATS) 1/2 and Mps one binder kinase activator (MOBs) (25). In the Hippo pathway, the phosphorylated MST1/2 and WW45 complexes activate LATS/MOB complex, and then phosphorylate YAP1, resulting in YAP1 translocation and degradation at the cytoplasm (26). The activated YAP1 enters the nucleus, and its protein structure is composed of TEA domain transcription factor (TEAD)-binding domain (TBD), 14-3-3 binding domain, two W-containing domain (WW) domains, coiled-coil (CC) domain, transactivation domain (TAD), and PDZ domain (27, 28). There is no DNA binding site in YAP1, and the downstream pathway is regulated by TEAD1 (**Figure 1**) (29).

YAP1 is considered to be the root of tumor and is generally activated in human malignant tumors. It is essential for the initiation and growth of most solid tumors, such as liver tumor (30). YAP1 is activated in the development and progression of liver tumor, which drives tumor cell survival, proliferation, invasive migration, metastasis, and stemness of liver tumor cells. It may also lead to resistance to chemotherapy, radiotherapy, and immunotherapy (31, 32). It is worth noting that YAP1 has different effects on tumor and peritumoral. Normal hepatocytes in liver tumor also show activation of YAP1. Still, the loss of YAP1 in hepatocytes around these tumors accelerates the growth of tumors, while activated YAP1 in peritumoral hepatocytes invades liver tumor (33). YAP1 is a potential target to improve the efficacy of ICIs in the treatment of liver tumor. Our previous research found that YAP1 suppression (knockdown or chemical inhibition) enhanced the effectiveness of anti-PD-1 immunotherapy in mice with liver tumors (34). This effect related to the suppression of immunosuppressive TME, the balance of intestinal microorganisms, and the homeostasis of lipid metabolism in HCC cells dependent on YAP1 (35–37).

Here, we review the vital role of YAP1 in liver tumor immune microenvironment and metabolic reprogramming, and discuss the current status of targeted YAP1 in treating liver tumor. We hope our research will provide potential evidence for YAP1-centric or combined immunotherapy.

## 2 The interaction between YAP1 and immune molecules in liver tumor niche

**Figure 2:**
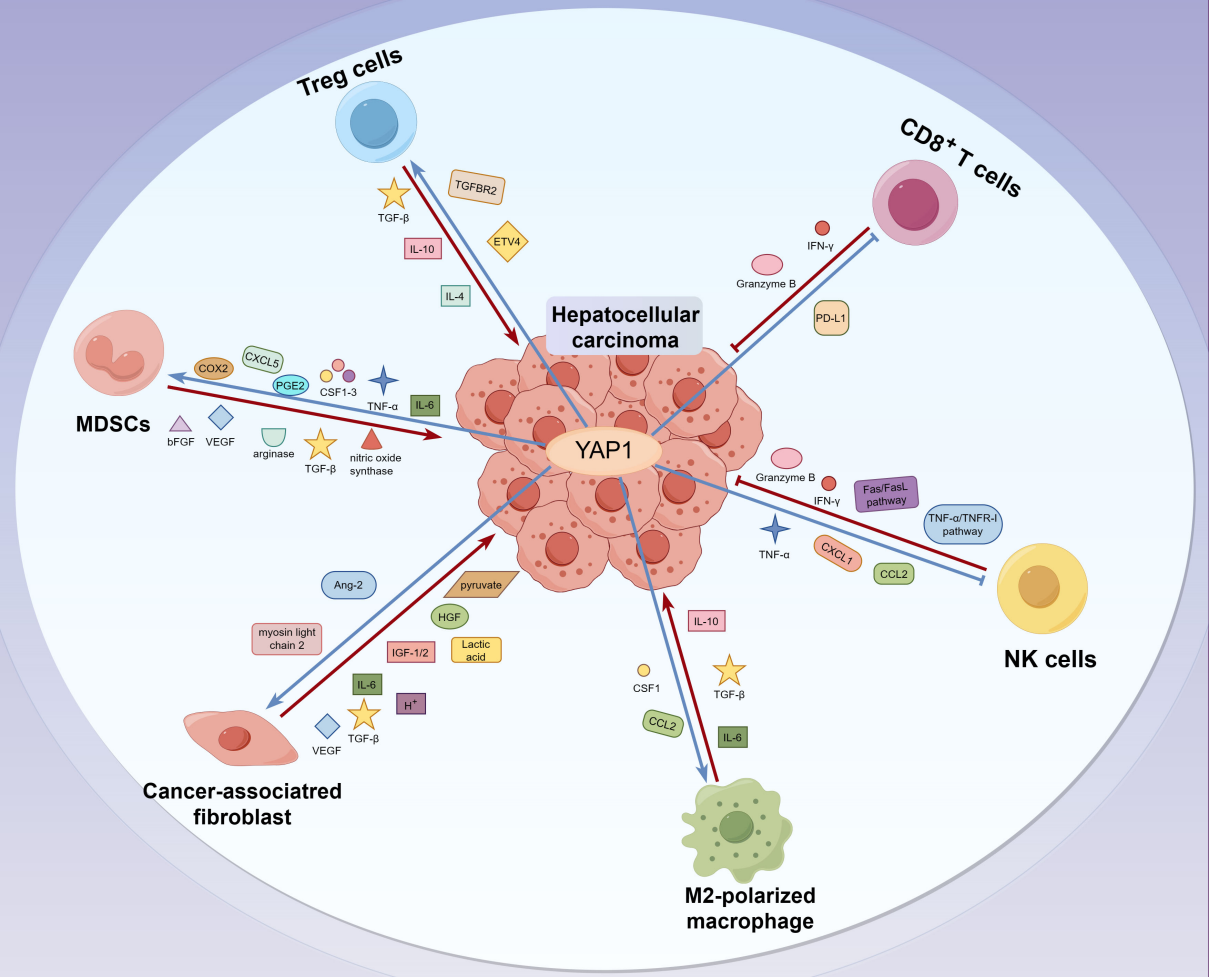
The interaction between YAP1 and immune molecules in TME of liver tumor. CD8^+^T and NK cells eliminate tumor cells by releasing IFN-γ and granzyme B. In contrast, Treg cells hinder the proliferation of effector T cells by releasing cytokines like TGF-β and IL-10, which creates an immunosuppressive TME that aids in the evasion of liver tumor cells. Moreover, MDSCs promote the escape and invasion of liver tumor cells by secreting immunosuppressive factors such as nitric oxide synthase, arginase, and TGF-β. Meanwhile, M2 macrophages secrete anti-inflammatory cytokines, including IL-10, IL-13, and IL-4, to inhibit immune clearance. In addition, CAFs, M2 macrophages, and MDSCs promote tumor metastasis by inducing the stiffening of the tumor ECM. Interestingly, YAP1 upregulates the infiltration of MDSCs, CAFs, M2-TAM, and Treg in liver tumor cells. Besides, YAP1 inhibits the activity of CD8^+^T and NK cells, promotes the formation of an immunosuppressive TME, and contributes to the evasion and progression of liver tumor cells.

TME is characterized by an anoxic and acidic environment, abnormal vascular proliferation, inflammation, and an immunosuppressive response, which plays a crucial role in tumor development and growth (38). Except for tumor cells, TME consists of stromal cells, such as tumor-associated fibroblasts (CAFs) and lymphocytes, including T cells, unconventional T cells, B cells, natural killer cells (NK), neutrophils, tumor-associated macrophages (TAMs), and myeloid-derived suppressor cells (MDSCs) in HCC. It also includes structural components like the extracellular matrix and intercellular communication-related molecules such as chemokines, cytokines, and exocrine substances (39, 40). These significantly impact tumor evasion, response to immunotherapy, and patient prognosis. The TME of liver tumor is characterized by the infiltration of tumor-infiltrating lymphocytes, including T cells, unconventional T cells, B cells, and NK. Unconventional T cells include natural killer T, (NKT), mucosal-associated T cells (MAIT), and γδT cells (41, 42). The anti-tumor immune response is mediated by cytotoxic CD8^+^T cells (CTLs), NK cells, NKT cells, γ δ T cells, and dendritic cells (DCs). M2-TAM, regulatory T cells (Tregs), MDSC, and CAFs encourage tumor cell immune evasion and hasten HCC spread (43). However, the current research does not involve the relationship between YAP1 and NKT cells, γ δ T cells or B cells in tumor immunity of HCC. YAP1 expression is inversely correlated with the prognosis of tumor patients (44). Here, we reviewed overexpressed YAP1 stimulates the production of cytokines, leading to increased infiltration of macrophages, MDSCs, and Tregs, ultimately resulting in an immunosuppressive TME that promotes tumor initiation and progression. Deficiency or inhibition of YAP1 enhances the killing ability of T cells and NK cells to tumor cells (45–50) (**Figure 2**).

### 2.1 T cells

T cells are the predominant immune cell subset observed in TME (51). One of the most significant immune surveillance cells is the CD8^+^T cells in the TME. A positive predictive mark in tumor tissue is a high abundance of CD8^+^T cells with killing capability. A high proportion of activated CD8^+^T cells in combination with low Tregs in the tumor niche has emerged as an independent prognostic factor for enhancing the overall survival rate and disease-free survival time in patients with HCC (52). It is beneficial to increase the percentage of CD8^+^T cells with a killing ability in tumor tissue to impede and even eradicate tumor growth (53, 54). CD8^+^T cells kill tumor cells by secreting enormous amounts of protease perforin, granzyme B, and interferon-gamma (IFN-γ) (55, 56). On the other hand, after CD8^+^T cells recognize tumor cells, the high level of FasL is expressed on the cell surface and bound to the Fas on the surface of the target cells, leading to programmed cell death in the tumor cells (57). However, ECM sclerosis caused by liver fibrosis reduced the infiltration of CD8^+^ T cells into HCC (58). The acidic and anoxic TME and the high proportion of Treg cells in HCC restrict the anti-tumor activity of CD8^+^T cells (59).

CD4^+^T cells promote anti-tumor immunity directly or indirectly through the help of CD8^+^T cells (60). Depletion of CD4^+^T cells diminished the anti-tumor effect (61). CD4^+^T cells are primarily categorized into Th1, Th2, Th9, Th17, Tfh, and Treg subsets based on their functions. Th1 cells assist CD8^+^T cells and secrete cytokines such as tumor necrosis factor-alpha (TNF-α), IFN-γ, and IL-2 to enhance the ability of CD8^+^T cells to target and kill tumor cells (62–64). Th2 cells are primarily involved in humoral immunity, secreting cytokines such as IL-4, IL-5, IL-6, and IL-10 to stimulate B cells to differentiate into plasma cells and produce antibodies, thereby maintaining the stability of humoral immunity (65). Th9 cells primarily participate in the immune response by secreting IL-9. Th17 cells mainly enhance the immune surveillance ability of the body and boost the immune response to liver tumor by secreting IL-17A/F, CC chemokine receptor 6 (CCR6), and other factors (64, 66). However, Th17 cells have also been reported to correlate positively with microvessel density in tumor tissues. This indicates that Th17 cells play a role in promoting angiogenesis and accelerating tumor progression (67). Tfh cells play a role in regulating humoral immunity. Treg cells mainly secrete cytokines such as transforming growth factor-beta (TGF-β), IL-4, and IL-10 to inhibit the proliferation of effector T cells or the proliferation of NK and effector T cells through direct cell-to-cell contact. The immunosuppressive environment created by Tregs promotes the evasion of tumor cells from detection and clearance by T cells, leading to the invasion and migration of tumor cells (57). The extent of tumor infiltration by Tregs was directly correlated with intra-tumoral vascular density, while the extent of CD8^+^T cell infiltration was inversely correlated. The high proportion of Treg cells reduces the survival rate of patients with HCC (68).

The high expression of YAP1 in tumor tissue can lead to the depletion of CD8^+^T and CD4^+^T cells, facilitating the proliferation and dissemination of liver tumor cells (36). In T cells activated by the TME, YAP1 played a role in immunosuppression and inhibition of effector differentiation. Loss of YAP1 in CD4^+^ and CD8^+^T cells enhanced T cell activation, differentiation, and cytotoxic function against tumor cells (49). In addition, verteporfin, an inhibitor of the binding YAP1 to TEAD, promoted T cell activation (69). However, neither the loss of YAP1 nor the use of inhibitors can regulate T cell proliferation. In addition to being highly expressed in numerous tumor cells, YAP1 is significantly expressed in Treg and CD8^+^T cells, inhibiting the immune response to tumors (50, 70). The expression level of YAP1 is upregulated in Tregs of peripheral blood mononuclear cells from patients with HCC. YAP1 fosters the differentiation of Tregs, particularly by enhancing the expression of TGF-β receptor 2, thereby promoting immunosuppression in the tumor niche (45). YAP1 was also expressed in activated CD8^+^T cells. YAP1 suppresses the anti-tumor response facilitated by suppressing the cytotoxicity of activated CD8^+^T cells (50). CD8^+^T cells lacking YAP1 produce more cytokines, such as IFN-γ and granzyme B in the niche (50). In summary, inhibiting the activity of YAP1 enhances the activation, differentiation, and cytotoxicity of T cells, hinders the differentiation of Tregs, and suppresses tumor growth.

### 2.2 NK cells

NK cells are considered the first line of defense against tumors. NK cells are a type of lymphocyte that can nonspecifically kill tumor cells without needing antigen presentation by MHC molecules (71). The main pathways through which NK cells resist tumor cells include the “missing self” mechanism and antibody-dependent cytotoxicity (ADCC). NK cells recognize and kill tumor cells (“missing self” phenotypic cells) that are not identifiable by T cells because of the down-regulation of MHC-I. The mechanism of ADCC involves NK cells recognizing B cell IgG-specific tumor cells and inducing target cell death by releasing perforin and granzyme or through the Fas/FasL pathway or TNF-α/TNFR-I pathway (71, 72). In addition to attacking tumor cells, NK cells also enhance the killing ability of T cells by releasing cytokines such as IFN-γ (72, 73).

Inhibition of LATS in the Hippo pathway increases the expression of YAP1 and PD-L1, suppressing the function of NK cells and promoting the apoptosis of T cells in tumors (74). Inhibition of YAP1 and STAT3 enhanced the killing effect of NK cells by reducing the expression of PD-L1 (75). The injection of NK cells can improve the death of tumor cells and inhibit the expression of YAP1, thereby reducing the tumor growth rate (76). In HCC, the interaction between the ETS transcription factor 4 (ETV4) and YAP1 activates the expression of CXCL1 and CC motif chemokine ligand 2(CCL2), leading to the infiltration of MDSCs and TAM while also down-regulating the number of T cells and NK cells. This interaction promotes the development and spread of HCC (77, 78). To sum up, the activity of YAP1 negatively regulates the number of NK cells and their killing effect. Inhibiting the expression of YAP1 is expected to become a new strategy to control the microenvironment of HCC.

### 2.3 MDSCs

MDSCs refer to a group of myeloid cells with immature characteristics and potent immunosuppressive function. Tumor cells stimulate the production of MDSCs by activating the JAK/STAT pathways (57). High levels of MDSC are associated with an increased risk of HCC and lower survival rates (79). On one hand, MDSCs inhibit T cell activity by secreting immunosuppressive factors, such as nitric oxide (NO) synthase and arginase. On the other hand, MDSC stimulates the production of immunosuppressive cells such as Treg and TAM (M2 type) by binding to TGF-β (57, 80). MDSC also inhibits the activation of CD8^+^T cells and liver NK cells and promotes the escape and infiltration of HCC cells (81). Furthermore, MDSCs promote tumor metastasis by producing vascular endothelial growth factor (VEGF), basic fibroblast growth factor (bFGF), and matrix metalloproteinases (MMP), which accelerate angiogenesis and enhance the stiffness of the extracellular matrix (ECM) (57).

YAP1 in tumors suppresses immunotherapy by upregulating the number of MDSCs (46, 82, 83). MDSC depletion caused by *YAP1* knockout enhances T cell activation and macrophage reprogramming (82). Activated YAP1 upregulates the secretion of IL-6 and TNF-α from tumor cells, as well as colony-stimulating factor 1-3 (CSF1-3), CXC motif chemokine ligand 5(CXCL5), PGE2, COX2, and other factors that recruit MDSCs (84). Verteporfin, the inhibitor of YAP1, downregulated IL-6, CSF1-3, and CXCL5, which compel MDSCs in tumor (85). Therefore, YAP1-TEAD complex creates an immunosuppressive microenvironment by stimulating tumor cells to secrete factors that recruit MDSCs, Tregs, and CAFs, which is conducive to the spread and invasion in tumor niche.

### 2.4 CAFs

The activation and widespread proliferation of CAFs is one of the characteristics of HCC (86). As an essential stromal cell in TME, CAFs are closely associated with the onset and progression of HCC. CAFs provide physical support for TME and secret proteins such as hepatocyte growth factor (HGF), and insulin-like growth factor-1/2 (IGF-1/2), among others in extracellular matrix (ECM) (87). HCC cells promote CAF proliferation, CAFs also promote the development and metastasis of HCC cells (88, 89). Further, CAFs inhibit the function of immune cells, promote tumor invasion and metastasis, and enhance the malignant phenotype of tumor cells through direct cell-to-cell contact, activation of cytokine secretion signal pathways, or increased production of metabolites (90, 91). CAFs secrete various cytokines, in which VEGF promotes angiogenesis, and TGF-β inhibits DC maturation and promotes Treg differentiation (92). IL-6 promotes MDSC differentiation and inhibits the function of CD8^+^T cells. CAFs also promote the solidification of tumor ECM by secreting large amounts of collagen and fibronectin. This hinders drug penetration or immune cells into tumor niche, reducing the therapeutic effect. Furthermore, exosomes derived from CAFs boost glycolysis, releasing substantial quantities of lactic acid and hydrogen ions. This process creates an acidic microenvironment that hinders the function of immune cells (93). A significant amount of ECM from CAFs increase the rigidity of the microenvironment and interstitial pressure, and promote tumor invasion and migration. Additionally, metabolites such as lactate and pyruvate produced by CAFs serve as nutrients for tumor cells, and support tumor cell metabolism.

The stiffness of ECM and cell-mediated tension play a role in mediating the contractility of cells. The interaction between YAP1 and TEAD is influenced by mechanical force (94). CAFs secrete MMP to remodel the ECM, increase tissue tension, and intensify the malignancy of tumor (93, 94). YAP1 enhances the activity of myosin light chain 2 to increase cell surface tension and regulate the activation of CAFs. The activated CAFs further improve the rigidity of ECM and maintain the high-tension state of CAFs (94). Furthermore, YAP1 is expressed in endothelial cells, and its activity is regulated by cell contact mediated by endothelial cell adhesion connexin and VE-cadherin. In turn, YAP1 regulates endothelial function by controlling the expression of ANG-2, which impacts tumor angiogenesis (95). YAP1-activated CAFs expressed higher levels of TGF- β and IL-4. In short, YAP1 was identified as a critical regulator of CAF activation; elevated levels of YAP1 are considered to activate CAFs, resulting in the stiffening of the ECM and the progression of the tumor (96). In turn, inhibiting the activity of YAP1 prevents the activation of CAFs (97). These findings are essential for inhibiting YAP1 to enhance the efficacy of HCC and regulate the tumor microenvironment.

### 2.5 TAMs

Tumor-associated macrophages include M1 and M2 subtypes. M1 macrophages secrete pro-inflammatory cytokines, such as IL-12, TNF-α, CXCL-10, and IFN-γ, and produce high levels of iNOS to eliminate tumor cells. M2 macrophages secrete anti-inflammatory cytokines, including IL-10, IL-13, and IL-4, to inhibit immune clearance. It also promotes tumor formation and growth by stimulating proliferation and angiogenesis (98, 99). Most TAMs exhibit an M2-like phenotype and secrete IL-10 and TGF-β to suppress tumor immunity in HCC (100). At the same time, IL-6 secreted by TAMs and activated STAT3 signaling promote the expansion of cancer stem cells (CSCs) and increase the progression and recurrence in liver tumor (101). The prognosis of patients with liver tumor was negatively correlated with the degree of M2 macrophage infiltration in the tumor niche (102).

The recruitment of tumor-initiating cell (TIC)-associated macrophages (TICAM) is crucial for tumor development and advancement. Overexpression of YAP1 and knockout of *Mst1/2* or *Lst1/2* recruit liver tumor initiation cells TICAM (46). In addition, it was found that activated YAP1 promotes the secretion of IL-6 by HCC cells, which subsequently encourages the chemotaxis and recruitment of TAMs in the liver (103). Using statins inhibits the expression of YAP1 and the accumulation of TAMs, while inhibiting or deleting IL-6 also demonstrates resistance to carcinogenesis (103–105). These results suggest that enhancing the infiltration of TAMs by regulating YAP1 could be a novel therapeutic strategy in HCC. Both CCL2 (also called MCP1) and CSF1 promote M2 polarization and contribute to tumor initiation and progression (106, 107). YAP1 activation upregulated the expression of CCL2 and CSF1. The transcriptional activity of YAP1/TEAD recruits TICAM, in which YAP1/TEAD1 directly binds to the promoter of *CCL2*. Oncogenes such as AKT/EGFR activate YAP1 in HCC, upregulate CCL2 expression, and recruit TICAM to promote the survival and tumorigenesis of TIC (46). However, another study suggests that *Mst1/2*-DKO mice activated YAP1 and increased CCL2 expression during HCC formation. This promoted M1 and M2 polarization and regulated the growth and proliferation of liver tumor cells (108). In summary, activated YAP1 recruits TAMs, promotes M2 polarization of macrophages, and accelerates the growth and proliferation of HCC cells.

## 3 YAP1 is extensively involved in the metabolic reprogramming of liver tumor

Metabolic reprogramming is a hallmark event in tumor cell, which is considered to drive the occurrence and progression of liver tumor (109). The enhancement of catabolism and anabolism of tumor cells mainly characterizes the metabolic reprogramming of tumor. This is usually due to adaptive changes caused by a lack of oxygen and insufficient nutrition in tumor cells (110). In recent years, with an in-depth understanding of the role of metabolism in the pathogenesis of liver tumor, regulating metabolic abnormalities is considered a potential new strategy for liver tumor treatment (111).

**Figure 3:**
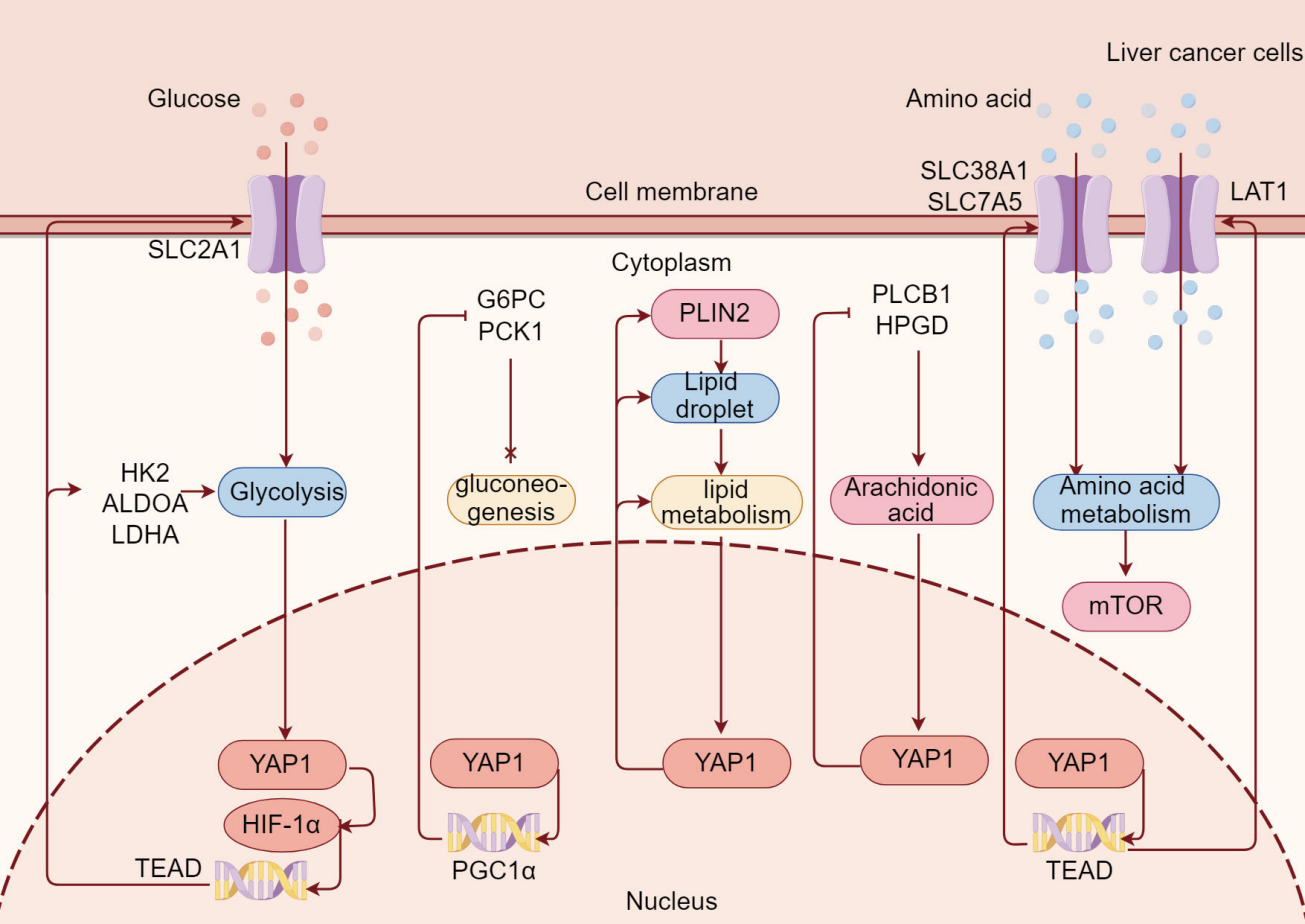
YAP1 is extensively involved in metabolic reprogramming in liver tumor. YAP1/TEAD binds to HIF-1α and promotes the transcription of the glycolysis gene, increases SLC2A1-mediated glucose uptake, and enhances glycolysis in liver tumor cells. Meanwhile, YAP1 promotes PGC1α transcription and inhibits G6PC and PCK1 to inhibit gluconeogenesis in liver tumor cells. At the same time, YAP1 enables PLIN2 on the surface of lipid droplets to improve lipid metabolism in liver tumor cells. Moreover, YAP1 inhibits PLCB1 and HPGD, thus promoting arachidonic acid metabolism in liver tumor cells. Besides, YAP1 promotes amino acid transporters SLC38A1, SLC7A5, and LAT1 through transcription factor TEAD, enhances amino acid uptake in liver tumor cells, and thus promotes mTOR.

YAP1 is involved in metabolism regulation and is an emerging node in coordinating nutrient availability with cell growth and tissue homeostasis (112). Indeed, YAP1 participates in the metabolic events of liver tumor cells such as glycolysis and lipid metabolism. Conversely, the state of glycolysis and lipid metabolism of liver tumor cells regulate YAP1. Currently, much is known about the regulation of YAP1 in metabolic reprogramming of tumor cells, but little is known about the regulation of YAP1 on metabolic reprogramming of immune cells in liver tumor. Here, we mainly review the important role of YAP1 in glycolysis, gluconeogenesis, lipid metabolism, arachidonic acid (AA) metabolism, and amino acid metabolism in liver tumor cells (**Figure 3**).

### 3.1 Aerobic glycolysis

Aerobic glycolysis is a crucial feature of glucose metabolism in tumor cells. Even under sufficient oxygen, tumor cells convert glucose to pyruvate and eventually produce lactic acid, characterized by increased glucose uptake and lactic acid production. This phenomenon was first discovered in HCC by Otto Warburg in the 1920s and named the Warburg effect (113). Aerobic glycolysis promotes the proliferation and growth of HCC (114). The rapid production of ATP during aerobic glycolysis makes the tumor cells adapt to the energy-deficient microenvironment. The glycolysis phenotype of liver tumor cells promotes the growth in HCC. This is due to the accumulation of lactic acid caused by aerobic glycolysis of tumor cells, which leads to the acidification of the tumor extracellular environment, inhibits the function of immune cells in TME, and leads to immune escape (115, 116). At the same time, enhancing aerobic glycolysis of liver tumor cells also promotes the invasion and metastasis through lactic acid-mediated extracellular acidification (117). Aerobic glycolysis causes angiogenesis of HCC in various ways, which further leads to rapid tumor growth and metastasis (118). In addition, aerobic glycolysis is an essential cause of drug resistance. Inhibition of glycolysis has been shown to improve the efficacy of sorafenib in HCC (119, 120).

There is increasing evidence that oncogenes and tumor suppressor genes regulate the abnormal glycolysis phenotype in HCC (113). The effective inhibition of aerobic glycolysis enhanced in liver tumor cells has a promising anti-tumor effect (121, 122). YAP1 has been demonstrated to be one of the most critical molecules in regulating glycolysis in HCC (123). As a tumor-promoting factor, YAP1 activation induced by hypoxia is the key to promoting glycolysis of HCC cells. Inhibition of YAP1 down-regulated glycolysis under hypoxia in HCC cells. Hypoxia stress encourages the binding of YAP1 and HIF-1α in the nucleus, directly activates pyruvate kinase M2 (PKM2) transcription, and finally accelerates the glycolysis phenotype in HCC cells (124). Even under normal oxygen conditions, YAP binds HIF-1α in the nucleus to activate the transcription of glycolysis genes, such as solute carrier family 2 member 1 (*SLC2A1*), hexokinase 2 *(HK2)*, aldolase A (*ALDOA*), and dehydrogenase A (*LDHA*). In turn, these proteins promote aerobic glycolysis in HCC cells and provide energy for tumor cell proliferation (125). In addition, our prior studies also suggested that *YAP1* knockdown/knockout reduced the SLC2A1-mediated Warburg effect in HepG2215 cells and mice with liver tumor induced by DEN/TCPOBOP (126).

It is reported that there is an interaction between YAP1 and metabolism (112). It is a remarkable fact that aerobic glycolysis of HCC cells, in turn, regulates YAP1 activity (127). Glycolysis is necessary to maintain the tumor-promoting function of YAP1, and YAP1 is needed to give full play to the growth-promoting activity of glucose. When the aerobic glycolysis was enhanced in HCC cells, the activity of YAP1 increased, and when the aerobic glycolysis of HCC cells was inhibited, the transcriptional activity of YAP1 decreased. The primary mechanism is phosphofructokinase (PFK1), which binds TEADs, a transcriptional cofactor of YAP1, and promotes their functional and biochemical synergism with YAP1 (128).

### 3.2 Gluconeogenic

Gluconeogenesis, the reverse pathway of glycolysis, syntheses glucose from non-carbohydrate substrates such as glycerol, lactate, pyruvate, and glucogenic amino acids (129, 130). The uncontrolled proliferation leads to excessive consumption of nutrients such as glucose, which leads to nutrient deprivation in tumor. The microenvironment of solid tumors is thought to be more prone to glucose deprivation, resulting in nutritional deficiencies (131). In the absence of glucose, tumor cells synthesize important metabolites through gluconeogenesis. However, gluconeogenesis promotes or inhibits tumors in different types of tumor (132).

Gluconeogenesis occurs mainly in hepatocytes and is considered to play an essential role in the tumor progression of HCC. Converting aerobic glycolysis of liver tumor cells to gluconeogenesis may be an effective strategy for treating HCC (133). Although the abbreviated form of gluconeogenesis helps tumor cells survive under glucose deficiency, gluconeogenesis reduces the production of glycolysis and lactic acid in HCC due to the rapid proliferation of HCC cells (134). Phosphoenolpyruvate carboxykinase 1 (PCK1) is the first rate-limiting enzyme of gluconeogenesis, and has an anti-tumor effect in liver tumor. PCK1 deficiency increases hepatic gluconeogenesis and promotes the proliferation of HCC (135).

Interestingly, YAP1 reprogrammed cell metabolism by transforming the substrate from energy-consuming gluconeogenesis to the anabolism of growth. YAP1 suppresses the expression of PCK1 by inhibiting the binding of peroxisome proliferator-activated receptor gamma coactivator 1 (PGC1α) to gluconeogenesis gene promoter, which leads to the activation of gluconeogenesis pathway and the progress of HCC. The inhibition of YAP1 induces the restriction of PCK1 on gluconeogenesis and restores the anti-tumor effect on HCC (136).

### 3.3 Lipid metabolism

Lipids serve vital biological roles within the human body, encompassing energy provision and storage, maintaining membrane structure and function, and signal transduction (137). In the context of tumor, aberrant lipid metabolism commonly occurs, characterized by heightened lipid metabolism during various stages of tumor progression. The surplus of lipids not only provides the energy supply for tumor cells but also fosters the proliferation and metastasis of these cells while instigating signal transduction and epigenetic occurrences (138, 139).

Lipids mainly comprise phospholipids, sphingolipids, triglycerides, fatty acids (FAs), and sterols. Phospholipids and sphingomyelins constitute the main components of the cell membrane’s lipid bilayer and are also involved in signal transduction. Triglycerides, including fat and oil, are the body’s primary forms of energy storage, mainly composed of FAs and glycerol. The main form of sterols in the body is cholesterol (137). Increasing evidence suggests that the increase of lipids, incredibly FAs, and cholesterol leads to a poor prognosis for patients with tumor. Although glycolysis is the primary metabolic mode of tumor cells, lipid-dependent metabolism is also an important energy source pathway for tumor cells. Tumor cells use FAs and cholesterol to meet excessive energy needs (140, 141). In addition, tumor cells synthesize FAs and activate them through covalent modification by fatty acyl-CoA synthetase, while the activated FAs are mainly stored in lipid droplets (LDs) (142).

The increase in lipid metabolism and the imbalance of lipid physiology often exist in HCC, resulting in abnormal lipid metabolism (143, 144). Increased fat production and imbalance of cholesterol biosynthesis are essential metabolic events in the development of HCC. Therefore, regulating abnormal lipid metabolism to target *de novo* FA synthesis and cholesterol biosynthesis is an important therapeutic strategy for HCC (145).

YAP1 induces lipid metabolism reprogramming in tumor cells (146). A comprehensive analysis of transcriptome and metabonomic in our study shows that *YAP1* knockdown inhibits the proliferation and metastasis mainly by regulating lipid metabolism in HCC cells (147). Our previous studies also show that *YAP1* knockdown/knockout reduces LD deposition and the membrane protein perilipin2 (PLIN2) expression on the surface of LDs in HCC cells (35, 148). Down-regulation of YAP1 reduces LD accumulation in mice with non-alcoholic fatty liver disease and inhibits the proliferation and invasion of HCC cells (149). In addition, YAP1 mediates the metabolic transformation of fatty acid oxidation (FAO) in tumor cells, which promotes tumor lymph node metastasis. Inhibition of YAP1 is a potential strategy to reduce tumor lymph node metastasis (150). Conversely, the expression or activity of YAP1 in HCC is also regulated by the lipid metabolism of tumor cells. Diacylglycerol lipase α (DAGLA) induces tumor cells to produce free FAs, enhances YAP1 activity, and aggravates the malignant phenotype and tumor progression in HCC (151). Statins, hydroxymethyl glutaryl-CoA reductase (HMGCR) inhibitor, reduce cholesterol biosynthesis and hypoxia-induced YAP1 activity in liver tumor cells (152). Therefore, there is an interaction between YAP1 and lipid metabolism in liver tumor cells. However, little is known about the regulatory role and mechanism of YAP1 on FA and cholesterol metabolism in HCC.

### 3.4 Arachidonic acid pathway

The arachidonic acid pathway is involved in the initiation, promotion, and progression of tumor. Arachidonic acid metabolic enzymes and their products regulate inflammation, cell proliferation, survival, angiogenesis, and invasion, thus promoting the occurrence and development of tumors (153). Previous studies have demonstrated that activation of arachidonic acid pathway promotes inflammation and tumorigenesis through cell, animal, and clinical evidence. Therefore, arachidonic acid metabolic enzymes phospholipase A2s (PLA2s), cyclooxygenases (COXs), and lipoxygenases (LOXs) and their metabolic products, such as prostaglandins and leukotrienes are considered potential targets for tumor therapy (154).

It has been noted that inflammation was intimately associated with the development and progression of HCC (155). Arachidonic acid metabolism promotes the progression of HCC by inducing more muscular liver inflammation (156). There is a strong correlation between the deregulation of arachidonic acid metabolism CYP450 pathway and the pathological features and prognosis of HCC (157). Furthermore, ethanol intake enhanced abnormal lipid metabolism induced by HBV through arachidonic acid pathway and activated Tregs in mice, which may lead to HCC (158). In a word, regulating the metabolism of arachidonic acid will be a promising way to treat HCC.

Recently, YAP1 promotes the metabolism of arachidonic acid by reverse-regulating phospholipase CB1 (PLCB1) and 15-hydroxyprostaglandin dehydrogenase (HPGD). Clinical evidence further suggests a potential correlation between abnormal activation of YAP1 and HCC progression induced by arachidonic acid metabolism. Aspirin shows therapeutic potential for HCC patients with abnormal YAP1 activation by regulating arachidonic acid metabolism (159).

### 3.5 Amino acid metabolism

Amino acids play tumorigenic and tumor-suppressive roles in tumor metabolism. Amino acids such as glutamic acid, branched-chain amino acids (BCAAs), and threonine provide fuel for tricarboxylic acid (TCA) cycle intermediates, and the release of ATP provides energy for oncogenic activities (160, 161). Amino acids support the biosynthesis of nucleotides, critical building materials for growth in tumor cells. Amino acids also affect the dynamic balance and epigenetic regulation of reactive oxygen species (ROS) through methylation and acetylation, and promote tumor invasiveness (162). Arginine metabolite supports tumor growth by promoting angiogenesis, and can also be used as a tumor suppressor (163). Furthermore, glutamine is the most critical type of amino acid in tumor nutrients because of its ability to convert nitrogen and carbon into growth-promoting pathways in tumor cells. Glutamine acts as an anaplerosis metabolite to drive the TCA cycle of tumor cells and produce ATP. Glutamine promotes the progression of malignant tumors by relaxing the control of energy, maintaining proliferation signals, achieving immortal replication, resisting cell death, and so on (164). Glutamine was activated by ECM stiffening in tumor cells and CAFs, increased the flow of non-essential amino acids and promoted the growth and invasiveness of tumor cells (165). Inhibition of amino acid metabolism is a potential therapy strategy for tumor metabolic pathways, but its intervention targets and the research and application of therapeutic drugs still face many challenges.

Liver tumor is particularly relevant in amino acid metabolism because the liver is the center of amino acid metabolism in human body (166, 167). The dynamic analysis of uptake and excretion flux of HepG2 cells shows that up to 30% of glutamine is metabolized in the cytoplasm, mainly used for nucleotide synthesis, cytoplasmic glutamate production, and cell growth maintenance. Partial inhibition of glutamate excretion inhibits the development of liver tumor cells (168). Particularly, amino acid metabolism-related genes (AAMRGs), are closely related to the prognosis of patients with HCC. Patients with high expression of AAMRGs in HCC patients have more abundant immunosuppressive cells and higher expression levels of suppressive immune checkpoints (169). BCAAs catabolism are related to HCC tumorigenesis. BCAA catabolism is activated in liver tumor cells without glutamine, and enhanced BCAA catabolism leads to BCAA-derived carbon and nitrogen flow toward nucleotide synthesis, stimulating cell-cycle progression and promoting cell survival in HCC cells. In summary, enhanced glutamine metabolism and BCAA catabolism promote the progress of HCC (170). Furthermore, amino acid transporters play an essential role in tumor progression and survival of HCC cells and can be used as emerging therapeutic targets (171, 172).

Our previous studies have shown that *YAP1* knockdown reduces amino acid metabolism in liver tumor cells (147). YAP1 is involved in glutamine metabolism reprogramming. YAP1 directly enhances the expression and activity of glutamine synthetase, increases the steady-state level of glutamine, and increases nucleotide biosynthesis, thus promoting the occurrence of liver tumor (173). Significantly, YAP1 mediates amino acid transport in liver tumor cells and participates in amino acid metabolism. YAP1 and transcriptional coactivator with PDZ-binding motif (TAZ) up-regulates the expression of amino acid transporter solute carrier family 38 member 1 (SLC38A1) and solute carrier family 7 member 5 (SLC7A5), increase amino acid uptake, activate mammalian rapamycin complex 1 (mTORC1), and stimulate the proliferation of HCC cells. The high expression of SLC38A1 and SLC7A5 was significantly correlated with the shorter survival time of the patients with HCC (29). Additionly, YAP1 induces the expression of leucine transporter LAT1 through transcription factor TEAD, a heterodimer complex of SLC7A5 and SLC3A2. Inhibition of YAP1 and reduction of LAT1 expression can reduce leucine uptake (174). Another study showed that YAP1 and NOTCH1 intracellular domain (NICD) were co-activated in mouse liver, which promoted the formation and rapid progression of ICC by activating amino acid transporter and mTOR1 (175).

## 4 Immunotherapy strategy of targeting YAP1 for liver tumor

It is well known that immunosuppression is a sign of tumor (176). The crosstalk between programmed cell death 1 (PD-1) and programmed cell death-ligand 1 (PD-L1) is one of the most well-studied and clinically successful drug targets in immune checkpoints (177). PD-L1 is on the surface of tumor cells, and causes the exhaustion of tumor-infiltrating CD8^+^T cells by the interaction of PD-1. Precisely,the binding of PD-1 to PD-L1 inhibits T cell proliferation and activation, promotes exhaustion, and initiates T cell apoptosis. Many of these phenomena can be reversed by blocking PD-1 or PD-L1 with monoclonal antibodies. As a result, the PD-1/PD-L1 axis has been considered an exciting therapeutic target for tumor therapy (178).

YAP1 translocation to the nucleus showed micron dot distribution in tumor cells. It was negatively co-located with heterochromatin in the infiltration area of CD8^+^T cells in different types of tumor tissues after immunotherapy. This is related to drug resistance in immunotherapy. When the initial tumor size is similar, the tumor produced by the YAP1 phase separation of defective cells is more sensitive to PD-1 therapy (179). It may be possible to use intrinsic YAP1 expression to screen tumor populations who may benefit from radiation therapy combined with immunotherapy. Patients with low expression levels of YAP1 will be more likely to benefit from anti-PD-1 therapy. Alternatively, patients with high expression levels of YAP1 should be considered for YAP1-targeted therapies (180).

The therapeutic effect of YAP1 inhibitor combined with ICIs is better than that of PD-1 monoclonal antibody alone (34, 35, 126, 181). Here, we summarized the existing drugs targeting YAP1 for liver tumor, including some drugs of natural origin (**Table 1**).

**Table 1:** Drugs targeting YAP1 for the treatment of liver tumor.

Drug	Highest phase	Functions	Source
Verteporfin	Pre-clinical	Suppress YAP1 function and hinder the progression of hepatocellular; Inhibit YAP1 by disrupting YAP1-TEAD interactions; Inhibit the growth of HCC through apoptosis mediated by mitochondrial dysfunction; Decrease the percentage of PD-1^+^CD8^+^T cells in the spleen of mice with liver tumor and increase the rate of PD-1^-^CD8^+^T cells in the peripheral blood of mice.	(36, 178)
Evodiamine	Pre-clinical	Promote LAST1 phosphorylation and inhibits YAP1 expression in HCC cells, resulting in YAP1 phosphorylation and reduced nuclear translocation; Induce apoptosis, inhibit tumor cell migration, invasion and epithelial-to-mesenchymal cell transition through YAP1.	(182, 183)
Decursin	Pre-clinical	Inhibit the expression of YAP1 in liver tumor cells by increasing LATS1 phosphorylation and beta-transducin repeat-containing protein (β-TRCP) expression.	(184)
WZ35	Pre-clinical	Inhibit the growth of liver tumor cells by down-regulating autophagy controlled through YAP1; Reduce SLC2A1-mediated glycolysis by inhibiting YAP1, thus inhibiting the proliferation of liver tumor cells.	(185, 186)
Dihydroartemisinin	Pre-clinical	Regulate lipid metabolism, TME, glycolysis, and intestinal microflora of the immune microenvironment through YAP1 in HCC, thus enhancing the effect of anti-PD-1 treatment.	(35–37, 126)
**Salvianolic acid B**	Pre-clinical	Inhibit the progression of liver tumor by adjusting Hippo/YAP pathway.	(187, 188)
Statins	Pre-clinical	Atto vastatin reduces the risk of liver tumor in patients with viral and metabolic liver disease and inhibits YAP1 activation; Simvastatin was used as a YAP1 inhibitor and was found to target YAP1 in combination with sorafenib or trametinib; Cerivastatin and simvastatin regulate the localization of YAP1 protein in HCC cells by inhibiting senescence junction and Rho GTP enzyme-mediated cytoskeleton remodeling involved in the migration and transfer of HCC.	(189, 190)

### 4.1 Verteporfin

Verteporfin is a recognized YAP1 inhibitor approved by the FDA for the treatment of neovascular macular degeneration (191). It has been identified as a small molecule inhibitor of TEAD-YAP1 association and inhibits YAP1 by disrupting YAP1-TEAD interactions (192, 193). It can suppress YAP1 function and hinder the progression of HCC (69). Verteporfin hinders subcutaneous graft tumor growth in the Huh-7 and Hepa1-6 cells of nude mice (125, 194). Verteporfin induces the permeability of tumor-specific lysosomal membranes after pH alkalinization in the lumen of the HCC cell line, causing a vital catabolism disorder, finally leading to an unsolvable intracellular protein toxicity (195).

YAP1 inhibitor verteporfin can inhibit the growth of HCC through apoptosis mediated by mitochondrial dysfunction (182). Verteporfin decreased the PD-1^+^CD8^+^T cell percentage while increasing the PD-1^-^CD8^+^T cell percentage in the spleen in liver tumor mice. TGF-β inhibits CD8^+^T cell activation and promotes Treg differentiation. Verteporfin decreased TGF-β levels in liver tumor mice (36). In addition, the transduction of oval cells with high activation of YAP1 in HCC gives tumorigenicity. The treatment of verteporfin on HCC model rats destroyed the formation of the YAP1-TEAD complex and significantly reduced pretumor lesions and oval cell proliferation. This suggests verteporfin-mediated YAP inhibition inhibited liver tumor cell growth (178). Notably, verteporfin also increases the sensitivity of HCC to sorafenib and reverses the drug resistance of sorafenib (196).

### 4.2 Evodiamine

Evodiamine is a quinazoline alkaloid, which is an effective ingredient isolated from the Chinese herbal medicine Wu Zhu Yu (*Evodia rutaecarpa (Juss.) Benth*) *(* 197). It has a variety of confirmed bioactivity, including anti-obesity, anti-inflammatory and anti-tumor effects (198–200). Evodiamine has antitumor activity in many kinds of tumors, such as lung tumor, gastric tumor, colorectal tumor and ovarian tumor (201–204). Previous studies have shown that evodiamine is a potential candidate for HCC therapy, which has the effect of anti-proliferation and inducing apoptosis, and inhibits the stem cell characteristics of HCC cells (205–207).

Recent studies have shown that evodiamine has become an effective anti-tumor drug in HCC by reducing the level of YAP1 (182, 183). Evodiamine promotes LAST1 phosphorylation and inhibits YAP1 expression in HCC cells, resulting in YAP1 phosphorylation and reduced nuclear translocation, thus inactivating its downstream effector molecules (182). Evodiamine decreased the expression of YAP1 and the growth of HCC cells in a dose-dependent manner, then induced apoptosis, inhibited tumor cell migration, invasion and epithelial-to-mesenchymal cell transition through YAP1 (183).

### 4.3 Decursin

Decursin was isolated from the roots of Dang Gui (*Angelica* g*igas Nakai*) (208). Pyranocoumarins, including decursinol, decursin, and decursinol angelate, are the main components identified in *A. gigas Nakai* (209). It shows high cytotoxicity in human - cell lines, whereas low cytotoxic activity in normal tissues indicates it may be a safe and attractive therapeutic medicament for tumor treatment (210). Decursin has been found to have potential therapeutic effects on malignant tumors such as gastric tumor, head and neck squamous cell carcinoma, human multiple myeloma, non-small cell lung tumor, bladder tumor and colon tumor (211–215). Notably, decursin shows anti-tumor effect by improving the activation of T cells in tumor microenvironment (210).

Recent studies have shown that decursin inhibits the proliferation of liver cells through apoptosis and cell cycle arrest, and its mechanism is related to the Hippo/YAP1 pathway (184). Mechanismly, decursin inhibits the expression of YAP1 in liver tumor cells by increasing LATS1 phosphorylation and beta-transducin repeat-containing protein (β-TRCP) expression. This leads to the phosphorylation and ubiquitin-mediated degradation of YAP1 protein, resulting in the inactivation of its downstream effectors (184). Importantly, decursin induced apoptosis of tumor cells can be reversed by selective MST1/2 inhibitors, which confirms that the antitumor effect of decursin in liver tumor depends on Hippo/YAP1 pathway (184).

### 4.4 WZ35

Curcumin is a natural polyphenol compound that is obtained and purified from the powdered rhizome of *Curcuma longa* L. (turmeric). Studies have shown that curcumin plays an important role in antibacterial, anti-proliferation, anti-inflammatory, anti-oxidation, anti-tumor, and anti-amyloidosis *in vitro* and *in vivo* by targeting various molecules (216). Curcumin inhibits cell proliferation, promotes apoptosis, inhibits tumor angiogenesis and metastasis, and induces autophagy in tumor cells (217, 218).

WZ35 is a new curcumin derivative, which shows anti-tumor activity in gastric tumor, breast tumor, colon tumor and prostate tumor (219–223). Recent studies have found that WZ35 has inhibitory activity in liver tumor, and its mechanism is related to the down-regulation of YAP1 expression (185, 186). On the one hand, WZ35 inhibits the growth of liver tumor cells by down-regulating autophagy controlled through YAP1. This inhibitory effect on autophagy of liver tumor cells is dependent on YAP1 (185). On the other hand, WZ35 reduces SLC2A1-mediated glycolysis by inhibiting YAP1, thus inhibiting the proliferation of liver tumor cells (186).

### 4.5 Dihydroartemisinin

Artemisinin comes from an annual member of Compositae *Artemisia annua*. As a traditional Chinese medicine with a history of more than 2000 years, dihydroartemisinin is not only the active metabolite of artemisinin and its derivatives (ART) but also the first-generation derivative of artemisinin. It is an effective medicine widely used to treat malaria (224, 225). In recent years, more and more attention has been paid to the antitumor activity of dihydroartemisinin. Dihydroartemisinin has been proven to have a strong anti-tumor effect and inhibit tumor cell proliferation *in vitro* and *in vivo*.

Our recent research shows that dihydroartemisinin directly inhibits YAP1 in HCC, and YAP1 serves as a cellular target of dihydroartemisinin in HCC cells. Previous studies of our group have shown that dihydroartemisinin regulates lipid metabolism, TME, and intestinal microflora of the immune microenvironment through YAP1 in HCC, thus enhancing the effect of anti-PD-1 treatment (35–37). Firstly, anti-PD-1 therapy promotes lipid drop (LD) deposition in HCC, which may lead to ineffective anti-PD-1 treatment. Inhibition of YAP1 decreased LD deposition and PLIN2 expression in HCC cells. Dihydroartemisinin reduced LD deposition and PLIN2 expression in HCC cells by inhibiting YAP1 (35). Secondly, dihydroartemisinin improves the immunosuppressive TME-induced immune escape by inhibiting YAP1, which leads to decreased PD-L1 expression in liver tumor cells and increased tumor-infiltrating CD8^+^T cells (36). Finally, anti-PD-1 therapy reduced the abundance of intestinal *Akkermansia muciniphila* (*A. muciniphila*) in HCC. Dihydroartemisinin increases the abundance of intestinal *A. muciniphila* and the number and activity of CD8^+^T cells in liver TME by inhibiting YAP1, thus enhancing the sensitivity of anti-PD-1 therapy (37). It is essential to underline that the effect of dihydroartemisinin on regulating lipid metabolism, TME, and intestinal microorganisms in enhancing the anti-PD-1 effect in HCC depends on YAP1. Additionly, dihydroartemisinin reduced SLC2A1-mediated glycolysis in HCC by inhibiting YAP1 (126). Studies have shown that glycolytic level in tumor negatively correlates with response to anti-PD1 therapy (226). Therefore, dihydroartemisinin may regulate glycolysis to improve the efficiency anti-PD-1 in HCC by inhibiting YAP1.

**Figure 4:**
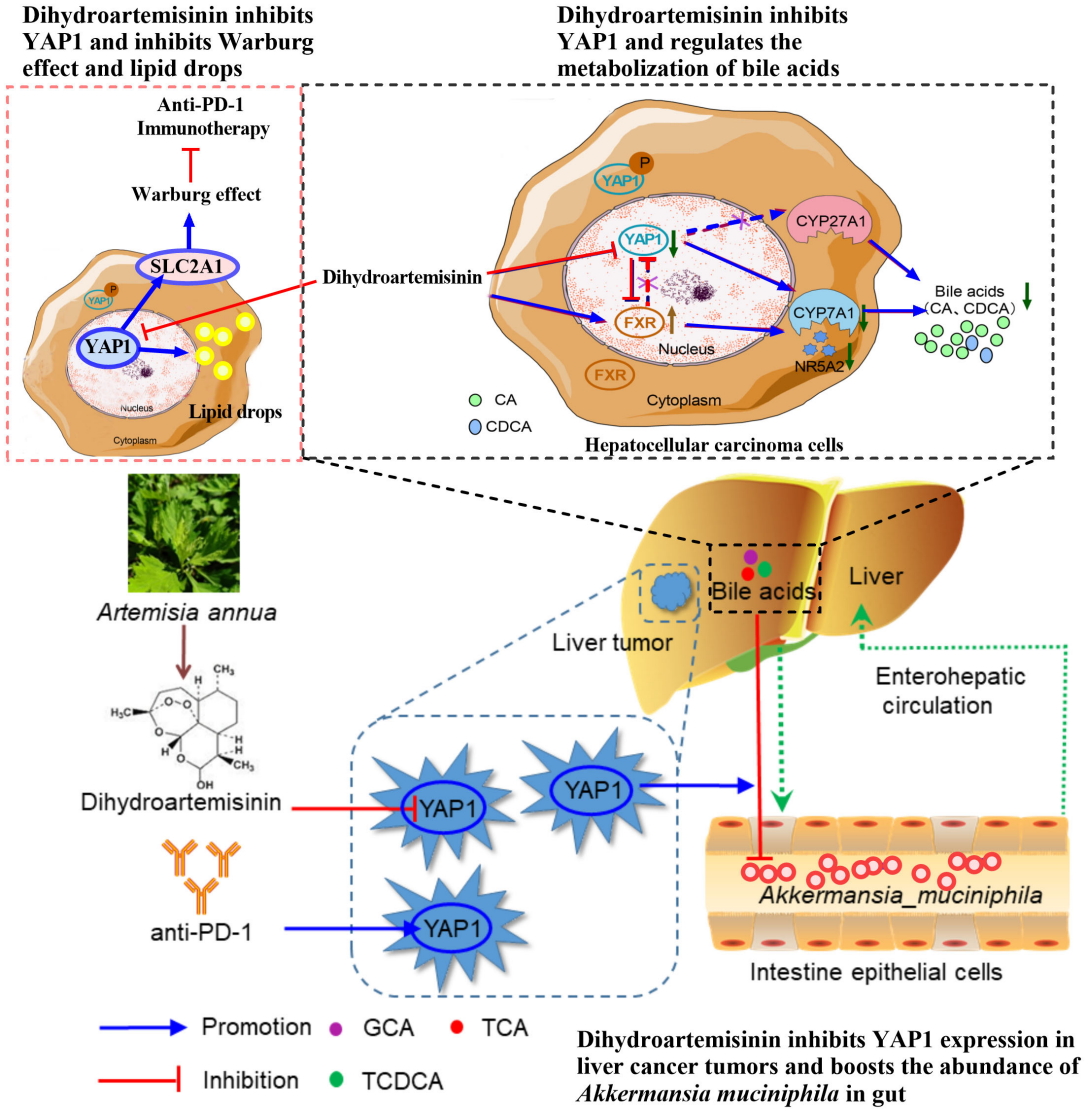
Dihydroartemisinin inhibits YAP1 expression and improves the anti-PD-1 effect in HCC. Dihydroartemisinin reduces the expression of glucose transporter SLC2A1 and lipid droplet deposition, regulates bile acid metabolism, and increases the abundance of *A. muciniphila* in the intestine by inhibiting YAP1 expression in HCC cells, leading to improvement of the therapeutic effect of anti-PD-1 (35–37, 126, 181).

In addition, dihydroartemisinin promoted farnesoid X receptor (FXR) and decreased cholesterol 7 α-hydroxylase (CYP7A1) and YAP1, thus inhibiting bile acid metabolism in HCC. However, the inhibitory effect of dihydroartemisinin on bile acid metabolism in HCC was independent of YAP1. In short, dihydroartemisinin has become a critical YAP1 inhibitor and a potential natural source of drugs based on immunotherapy to improve the efficacy of anti-PD-1 in HCC. The mechanism that dihydroartemisinin depends on YAP1 to enhance the sensitivity of anti-PD-1 in HCC still needs to be further elucidated (181) (**Figure 4**).

### 4.6 Salvianolic acid B

Salvianolic acid B (Sal B) is a major phenolic compound derived from the root and rhizome of *Salvia miltiorrhiza Bunge* (Labiatae) (227, 228). Sal B has extensive therapeutic effects on tumors, atherosclerosis, inflammation, and oxidative stress (203), and is recognized as a valid treatment for liver disease. Sal B relieve liver fibrosis in patients with HBV with no apparent side effects and favorably impact liver fibrosis in animal models. It also inhibits the proliferation and activation of hepatic stellate cells stimulated by TGF-β (227, 229). Sal B also improved atherosclerosis via inhibiting the YAP/TAZ/JNK signaling pathway. Previous studies have shown that SalB inhibits the occurrence of HCC by inducing apoptosis in tumor cells (187).

Sal B inhibits the progression of liver tumor and suppress the growth of liver tumor cells by inhibiting cell proliferation and migration. The mechanism is related to adjusting the Hippo/YAP pathway and simultaneously promoting linker-phosphorylated Smad3 (pSmad3L) to C-terminally phosphorylated Smad3 (pSmad3C) transformation (188).

### 4.7 Statins

Statins are first-line drugs for the treatment of hypercholesterolemia. They have been widely used since the end of the 20th century and are now one of the most commonly abused prescription drugs in the world (230). FDA has approved seven statins: lovastatin, pivastatin, Atto vastatin, rosuvastatin, pravastatin, simvastatin and fluvastatin (231). Increasing evidence emphasizes the importance of statins and reducing the risk of HCC (232). Lipophilic statins significantly reduced the morbidity and mortality of HCC in patients with viral hepatitis, and the use of dose-dependent statins significantly reduced the incidence of HCC in patients with nonalcoholic steatohepatitis (NASH) and liver cirrhosis (233, 234). Atto vastatin is considered to be the most effective statin to reduce the risk of liver tumor in patients with viral and metabolic liver disease, and its mechanism is related to the significant inhibition of YAP1 activation. Atto vastatin promotes YAP1 phosphorylation at two sites of Ser127 and Ser397 and reduces nuclear translocation of YAP1 (235). Simvastatin was used as a YAP1 inhibitor and was found to target YAP1 in combination with sorafenib or trimetinib. Simvastatin showed potent synergistic cytotoxicity in HCC cells and improved the therapeutic effect of sorafenib or trimetinib in HCC (189). In addition, statins also reduce RhoA activity by inhibiting YAP1 activity. Cerivastatin and simvastatin regulate the localization of YAP1 protein in HCC cells by inhibiting senescence junction and Rho GTP enzyme-mediated cytoskeleton remodeling involved in the migration and transfer of HCC (190, 234, 236). It is noteworthy that simvastatin has also been shown to synergize with anti-PD-L1 in tumor by inhibiting the mevalonate pathway, and the mechanism may be related to YAP1 (237).

## 5 Conclusions

YAP1 is a highly relevant factor in all stages of liver tumor, and we underline the central importance of YAP1 expression in ICI treatment of liver tumor. YAP1 promotes MDSCs, CAFs, M2-TAM, and Treg infiltration in liver tumor cells and inhibits the activity of CD8^+^T and NK cells. Meanwhile, YAP1 mediates metabolic reprogramming of liver tumor cells, enhances aerobic glycolysis, lipid metabolism, arachidonic acid metabolism, amino acid metabolism, and reduces gluconeogenesis. These YAP1-induced changes in tumor microenvironment and metabolic reprogramming promote the progression of HCC and the inefficacy of ICI therapy. After reviewing the current literature, we proposed that YAP1 inhibition is a treatment option to improve the efficacy of ICIs for more patients with advanced liver tumor. The previous findings emphasize the significance of YAP1 suppression in ICIs for liver tumor, in which YAP1 induces immunosuppressive TME and is involved in the metabolic reprogramming of liver tumor cells.

Due to YAP1 plays multiple roles in different cells of liver tumor, including tumor cells and immune cells, there will be a continuing important role for YAP1 in the field of tumor immunology, especially in the exploration of tumor immunotherapy. Inhibition of YAP1 by knockdown or chemical inhibitors in liver tumor has been proven to have the effect of sensitizing ICIs. This effect is related to improving liver tumor immunosuppressive TME and recovering abnormal metabolic reprogramming of liver tumor cells. It has been proven to be dependent on YAP1. Several drugs that inhibit YAP1 in HCC have been mentioned, especially Verteporfin, which destroys the YAP1-TEAD complex, and, DHA, a derivative from artemisinin. Although the relevance of YAP1 and the YAP1 inhibitors on liver tumor cells is already known, this knowledge has not yet been combined in clinical studies. This opens up a vast potential for future investigations into a possible cotherapeutic of YAP1 inhibitors in liver tumor. However, the key to applying the combined therapy strategy to patients with liver tumor is to find safe and effective YAP1 inhibitors and clarify the mechanism. Expanding future research questions can be worthwhile, primarily by transferring preclinical knowledge from the bench to the bedside.

## Author contributions

YtG: Conceptualization, Writing – original draft. YG: Writing – original draft. JL: Writing – original draft. HH: Writing – review & editing. XS: Writing – review & editing.

## References

[1] Siegel RL Miller KD Jemal A. Cancer statistics, 2019. CA Cancer J Clin. (2019) 69:7–34. doi: 10.3322/caac.21551 30620402

[2] Sung H Ferlay J Siegel RL Laversanne M Soerjomataram I Jemal A. Global cancer statistics 2020: globocan estimates of incidence and mortality worldwide for 36 cancers in 185 countries. CA Cancer J Clin. (2021) 71:209–49. doi: 10.3322/caac.21660 33538338

[3] Chaisaingmongkol J Budhu A Dang H Rabibhadana S Pupacdi B Kwon SM. Common molecular subtypes among Asian hepatocellular carcinoma and cholangiocarcinoma. Cancer Cell. (2017) 32:57–70.e3. doi: 10.1016/j.ccell.2017.05.009 28648284 PMC5524207

[4] Starley BQ Calcagno CJ Harrison SA. Nonalcoholic fatty liver disease and hepatocellular carcinoma: A weighty connection. Hepatology. (2010) 51:1820–32. doi: 10.1002/hep.23594 20432259

[5] Center MM Jemal A. International trends in liver cancer incidence rates. Cancer Epidemiol Biomarkers Prev. (2011) 20:2362–8. doi: 10.1158/1055-9965.Epi-11-0643 21921256

[6] Huang DQ Singal AG Kono Y Tan DJH El-Serag HB Loomba R. Changing global epidemiology of liver cancer from 2010 to 2019: nash is the fastest growing cause of liver cancer. Cell Metab. (2022) 34:969–77.e2. doi: 10.1016/j.cmet.2022.05.003 35793659 PMC9762323

[7] Brown ZJ Tsilimigras DI Ruff SM Mohseni A Kamel IR Cloyd JM. Management of hepatocellular carcinoma: A review. JAMA Surg. (2023) 158:410–20. doi: 10.1001/jamasurg.2022.7989 36790767

[8] Xu Q Li Y Gao X Kang K Williams JG Tong L. Hnf4α Regulates sulfur amino acid metabolism and confers sensitivity to methionine restriction in liver cancer. Nat Commun. (2020) 11:3978. doi: 10.1038/s41467-020-17818-w 32770044 PMC7414133

[9] Wang YG Wang T Shi M Zhai B. Long noncoding rna epb41l4a-as2 inhibits hepatocellular carcinoma development by sponging mir-301a-5p and targeting foxl1. J Exp Clin Cancer Res. (2019) 38:153. doi: 10.1186/s13046-019-1128-9 30971290 PMC6458726

[10] Reig M Forner A Rimola J Ferrer-Fàbrega J Burrel M Garcia-Criado Á. Bclc strategy for prognosis prediction and treatment recommendation: the 2022 update. J Hepatol. (2022) 76:681–93. doi: 10.1016/j.jhep.2021.11.018 PMC8866082 34801630

[11] El-Serag HB Marrero JA Rudolph L Reddy KR. Diagnosis and treatment of hepatocellular carcinoma. Gastroenterology. (2008) 134:1752–63. doi: 10.1053/j.gastro.2008.02.090 18471552

[12] Anwanwan D Singh SK Singh S Saikam V Singh R. Challenges in liver cancer and possible treatment approaches. Biochim Biophys Acta Rev Cancer. (2020) 1873:188314. doi: 10.1016/j.bbcan.2019.188314 31682895 PMC6981221

[13] Llovet JM Castet F Heikenwalder M Maini MK Mazzaferro V Pinato DJ. Immunotherapies for hepatocellular carcinoma. Nat Rev Clin Oncol. (2022) 19:151–72. doi: 10.1038/s41571-021-00573-2 34764464

[14] Donne R Lujambio A. The liver cancer immune microenvironment: therapeutic implications for hepatocellular carcinoma. Hepatology. (2023) 77:1773–96. doi: 10.1002/hep.32740 PMC9941399 35989535

[15] Xu F Jin T Zhu Y Dai C. Immune checkpoint therapy in liver cancer. J Exp Clin Cancer Res. (2018) 37:110. doi: 10.1186/s13046-018-0777-4 29843754 PMC5975687

[16] Yang C Zhang H Zhang L Zhu AX Bernards R Qin W. Evolving therapeutic landscape of advanced hepatocellular carcinoma. Nat Rev Gastroenterol Hepatol. (2023) 20:203–22. doi: 10.1038/s41575-022-00704-9 36369487

[17] Oura K Morishita A Tani J Masaki T. Tumor immune microenvironment and immunosuppressive therapy in hepatocellular carcinoma: A review. Int J Mol Sci. (2021) 22(11):5801. doi: 10.3390/ijms22115801 34071550 PMC8198390

[18] Wang Z Wang Y Gao P Ding J. Immune checkpoint inhibitor resistance in hepatocellular carcinoma. Cancer Lett. (2023) 555:216038. doi: 10.1016/j.canlet.2022.216038 36529238

[19] Zongyi Y Xiaowu L. Immunotherapy for hepatocellular carcinoma. Cancer Lett. (2020) 470:8–17. doi: 10.1016/j.canlet.2019.12.002 31811905

[20] Weng CY Kao CX Chang TS Huang YH. Immuno-metabolism: the role of cancer niche in immune checkpoint inhibitor resistance. Int J Mol Sci. (2021) 22(3):1258. doi: 10.3390/ijms22031258 33514004 PMC7865434

[21] Foglia B Beltrà M Sutti S Cannito S. Metabolic reprogramming of hcc: A new microenvironment for immune responses. Int J Mol Sci. (2023) 24(8):7463. doi: 10.3390/ijms24087463 37108625 PMC10138633

[22] Xia L Oyang L Lin J Tan S Han Y Wu N. The cancer metabolic reprogramming and immune response. Mol Cancer. (2021) 20:28. doi: 10.1186/s12943-021-01316-8 33546704 PMC7863491

[23] Zhang J Zheng Y Wang Y Wang J Sang A Song X. Yap1 alleviates sepsis-induced acute lung injury *via* inhibiting ferritinophagy-mediated ferroptosis. Front Immunol. (2022) 13:884362. doi: 10.3389/fimmu.2022.884362 35979359 PMC9376389

[24] Wu H Liu Y Liao Z Mo J Zhang Q Zhang B. The role of yap1 in liver cancer stem cells: proven and potential mechanisms. biomark Res. (2022) 10:42. doi: 10.1186/s40364-022-00387-z 35672802 PMC9171972

[25] Pan D. The hippo signaling pathway in development and cancer. Dev Cell. (2010) 19:491–505. doi: 10.1016/j.devcel.2010.09.011 20951342 PMC3124840

[26] Zhao B Li L Lei Q Guan KL. The hippo-yap pathway in organ size control and tumorigenesis: an updated version. Genes Dev. (2010) 24:862–74. doi: 10.1101/gad.1909210 PMC2861185 20439427

[27] Piccolo S Dupont S Cordenonsi M. The biology of yap/taz: hippo signaling and beyond. Physiol Rev. (2014) 94:1287–312. doi: 10.1152/physrev.00005.2014 25287865

[28] Cunningham R Hansen CG. The hippo pathway in cancer: yap/taz and tead as therapeutic targets in cancer. Clin Sci (Lond). (2022) 136:197–222. doi: 10.1042/cs20201474 35119068 PMC8819670

[29] Park YY Sohn BH Johnson RL Kang MH Kim SB Shim JJ. Yes-associated protein 1 and transcriptional coactivator with pdz-binding motif activate the mammalian target of rapamycin complex 1 pathway by regulating amino acid transporters in hepatocellular carcinoma. Hepatology. (2016) 63:159–72. doi: 10.1002/hep.28223 PMC4881866 26389641

[30] Zanconato F Cordenonsi M Piccolo S. Yap/taz at the roots of cancer. Cancer Cell. (2016) 29:783–803. doi: 10.1016/j.ccell.2016.05.005 27300434 PMC6186419

[31] Thompson BJ. Yap/taz: drivers of tumor growth, metastasis, and resistance to therapy. Bioessays. (2020) 42:e1900162. doi: 10.1002/bies.201900162 32128850

[32] Qing J Ren Y Zhang Y Yan M Zhang H Wu D. Dopamine receptor D2 antagonism normalizes profibrotic macrophage-endothelial crosstalk in non-alcoholic steatohepatitis. J Hepatol. (2022) 76:394–406. doi: 10.1016/j.jhep.2021.09.032 34648896

[33] Moya IM Castaldo SA Van den Mooter L Soheily S Sansores-Garcia L Jacobs J. Peritumoral activation of the hippo pathway effectors yap and taz suppresses liver cancer in mice. Science. (2019) 366:1029–34. doi: 10.1126/science.aaw9886 31754005

[34] Gao Y Peng Q Li S Zheng K Gong Y Xue Y. Yap1 suppression inhibits autophagy and improves the efficacy of anti-pd-1 immunotherapy in hepatocellular carcinoma. Exp Cell Res. (2023) 424:113486. doi: 10.1016/j.yexcr.2023.113486 36693491

[35] Hao L Guo Y Peng Q Zhang Z Ji J Liu Y. Dihydroartemisinin reduced lipid droplet deposition by yap1 to promote the anti-pd-1 effect in hepatocellular carcinoma. Phytomedicine. (2022) 96:153913. doi: 10.1016/j.phymed.2021.153913 35026515

[36] Peng Q Li S Shi X Guo Y Hao L Zhang Z. Dihydroartemisinin broke the tumor immunosuppressive microenvironment by inhibiting yap1 expression to enhance anti-pd-1 efficacy. Phytother Res. (2023) 37:1740–53. doi: 10.1002/ptr.7695 36576358

[37] Zhang Z Shi X Ji J Guo Y Peng Q Hao L. Dihydroartemisinin increased the abundance of akkermansia muciniphila by yap1 depression that sensitizes hepatocellular carcinoma to anti-pd-1 immunotherapy. Front Med. (2023) 17:729–46. doi: 10.1007/s11684-022-0978-2 37121958

[38] Luo N. Editorial: tumor microenvironment in cancer hallmarks and therapeutics. Front Mol Biosci. (2022) 9:1019830. doi: 10.3389/fmolb.2022.1019830 36172048 PMC9511906

[39] Li X Yang Y Huang Q Deng Y Guo F Wang G. Crosstalk between the tumor microenvironment and cancer cells: A promising predictive biomarker for immune checkpoint inhibitors. Front Cell Dev Biol. (2021) 9:738373. doi: 10.3389/fcell.2021.738373 34692696 PMC8529050

[40] Marie-Egyptienne DT Lohse I Hill RP. Cancer stem cells, the epithelial to mesenchymal transition (Emt) and radioresistance: potential role of hypoxia. Cancer Lett. (2013) 341:63–72. doi: 10.1016/j.canlet.2012.11.019 23200673

[41] Kurioka A Klenerman P. Aging unconventionally: Γδ T cells, inkt cells, and mait cells in aging. Semin Immunol. (2023) 69:101816. doi: 10.1016/j.smim.2023.101816 37536148 PMC10804939

[42] Constantinides MG Belkaid Y. Early-life imprinting of unconventional T cells and tissue homeostasis. Science. (2021) 374:eabf0095. doi: 10.1126/science.abf0095 34882451 PMC8697520

[43] Li Z Zhang Z Fang L Zhao J Niu Z Chen H. Tumor microenvironment composition and related therapy in hepatocellular carcinoma. J Hepatocell Carcinoma. (2023) 10:2083–99. doi: 10.2147/jhc.S436962 PMC10676104 38022729

[44] Wu Y Hou Y Xu P Deng Y Liu K Wang M. The prognostic value of yap1 on clinical outcomes in human cancers. Aging (Albany NY). (2019) 11:8681–700. doi: 10.18632/aging.102358 PMC6814621 31613226

[45] Fan Y Gao Y Rao J Wang K Zhang F Zhang C. Yap-1 promotes tregs differentiation in hepatocellular carcinoma by enhancing tgfbr2 transcription. Cell Physiol Biochem. (2017) 41:1189–98. doi: 10.1159/000464380 28472799

[46] Guo X Zhao Y Yan H Yang Y Shen S Dai X. Single tumor-initiating cells evade immune clearance by recruiting type ii macrophages. Genes Dev. (2017) 31:247–59. doi: 10.1101/gad.294348.116 PMC5358722 28223311

[47] Lee BS Park DI Lee DH Lee JE Yeo MK Park YH. Hippo effector yap directly regulates the expression of pd-L1 transcripts in egfr-tki-resistant lung adenocarcinoma. Biochem Biophys Res Commun. (2017) 491:493–9. doi: 10.1016/j.bbrc.2017.07.007 28684311

[48] Wang G Lu X Dey P Deng P Wu CC Jiang S. Targeting yap-dependent mdsc infiltration impairs tumor progression. Cancer Discovery. (2016) 6:80–95. doi: 10.1158/2159-8290.Cd-15-0224 26701088 PMC4707102

[49] Stampouloglou E Cheng N Federico A Slaby E Monti S Szeto GL. Yap suppresses T-cell function and infiltration in the tumor microenvironment. PloS Biol. (2020) 18:e3000591. doi: 10.1371/journal.pbio.3000591 31929526 PMC6980695

[50] Lebid A Chung L Pardoll DM Pan F. Yap attenuates cd8 T cell-mediated anti-tumor response. Front Immunol. (2020) 11:580. doi: 10.3389/fimmu.2020.00580 32322254 PMC7158852

[51] Pearce H Croft W Nicol SM Margielewska-Davies S Powell R Cornall R. Tissue-resident memory T cells in pancreatic ductal adenocarcinoma coexpress pd-1 and tigit and functional inhibition is reversible by dual antibody blockade. Cancer Immunol Res. (2023) 11:435–49. doi: 10.1158/2326-6066.Cir-22-0121 PMC10068448 36689623

[52] Gao Q Qiu SJ Fan J Zhou J Wang XY Xiao YS. Intratumoral balance of regulatory and cytotoxic T cells is associated with prognosis of hepatocellular carcinoma after resection. J Clin Oncol. (2007) 25:2586–93. doi: 10.1200/jco.2006.09.4565 17577038

[53] Naito Y Saito K Shiiba K Ohuchi A Saigenji K Nagura H. Cd8+ T cells infiltrated within cancer cell nests as a prognostic factor in human colorectal cancer. Cancer Res. (1998) 58:3491–4. 9721846

[54] Wang B Wang Y Sun X Deng G Huang W Wu X. Cxcr6 is required for antitumor efficacy of intratumoral cd8(+) T cell. J Immunother Cancer. (2021) 9(8):e003100. doi: 10.1136/jitc-2021-003100 34462326 PMC8407215

[55] Jia L Wang Y Wang CY. Circfat1 promotes cancer stemness and immune evasion by promoting stat3 activation. Adv Sci (Weinh). (2021) 8:2003376. doi: 10.1002/advs.202003376 34258151 PMC8261519

[56] Hinrichs CS Kaiser A Paulos CM Cassard L Sanchez-Perez L Heemskerk B. Type 17 cd8+ T cells display enhanced antitumor immunity. Blood. (2009) 114:596–9. doi: 10.1182/blood-2009-02-203935 PMC2713473 19471017

[57] Chen Y Zhou Y Yan Z Tong P Xia Q He K. Effect of infiltrating immune cells in tumor microenvironment on metastasis of hepatocellular carcinoma. Cell Oncol (Dordr). (2023) 46:1595–604. doi: 10.1007/s13402-023-00841-6 PMC12974623 37414962

[58] Guidotti LG Inverso D Sironi L Di Lucia P Fioravanti J Ganzer L. Immunosurveillance of the liver by intravascular effector cd8(+) T cells. Cell. (2015) 161:486–500. doi: 10.1016/j.cell.2015.03.005 25892224 PMC11630812

[59] Fu J Xu D Liu Z Shi M Zhao P Fu B. Increased regulatory T cells correlate with cd8 T-cell impairment and poor survival in hepatocellular carcinoma patients. Gastroenterology. (2007) 132:2328–39. doi: 10.1053/j.gastro.2007.03.102 17570208

[60] Ventura A Vassall A Robinson E Filler R Hanlon D Meeth K. Extracorporeal photochemotherapy drives monocyte-to-dendritic cell maturation to induce anticancer immunity. Cancer Res. (2018) 78:4045–58. doi: 10.1158/0008-5472.Can-18-0171 29764863

[61] Older SA Battafarano DF Danning CL Ward JA Grady EP Derman S. The effects of delta wave sleep interruption on pain thresholds and fibromyalgia-like symptoms in healthy subjects; correlations with insulin-like growth factor I. J Rheumatol. (1998) 25:1180–6. 9632083

[62] Melssen M Slingluff CL Jr. Vaccines targeting helper T cells for cancer immunotherapy. Curr Opin Immunol. (2017) 47:85–92. doi: 10.1016/j.coi.2017.07.004 28755541 PMC5757837

[63] Borst J Ahrends T Bąbała N Melief CJM Kastenmüller W. Cd4(+) T cell help in cancer immunology and immunotherapy. Nat Rev Immunol. (2018) 18:635–47. doi: 10.1038/s41577-018-0044-0 30057419

[64] Kim HJ Cantor H. Cd4 T-cell subsets and tumor immunity: the helpful and the not-so-helpful. Cancer Immunol Res. (2014) 2:91–8. doi: 10.1158/2326-6066.Cir-13-0216 24778273

[65] Wan S Ni L Zhao X Liu X Xu W Jin W. Costimulation molecules differentially regulate the erk-zfp831 axis to shape T follicular helper cell differentiation. Immunity. (2021) 54:2740–55.e6. doi: 10.1016/j.immuni.2021.09.018 34644536

[66] Qiu W Wang B Gao Y Tian Y Tian M Chen Y. Targeting histone deacetylase 6 reprograms interleukin-17-producing helper T cell pathogenicity and facilitates immunotherapies for hepatocellular carcinoma. Hepatology. (2020) 71:1967–87. doi: 10.1002/hep.30960 31539182

[67] Zhang JP Yan J Xu J Pang XH Chen MS Li L. Increased intratumoral il-17-producing cells correlate with poor survival in hepatocellular carcinoma patients. J Hepatol. (2009) 50:980–9. doi: 10.1016/j.jhep.2008.12.033 19329213

[68] Huang Y Wang FM Wang T Wang YJ Zhu ZY Gao YT. Tumor-infiltrating foxp3+ Tregs and cd8+ T cells affect the prognosis of hepatocellular carcinoma patients. Digestion. (2012) 86:329–37. doi: 10.1159/000342801 23207161

[69] Liu-Chittenden Y Huang B Shim JS Chen Q Lee SJ Anders RA. Genetic and pharmacological disruption of the tead-yap complex suppresses the oncogenic activity of yap. Genes Dev. (2012) 26:1300–5. doi: 10.1101/gad.192856.112 PMC3387657 22677547

[70] Ni X Tao J Barbi J Chen Q Park BV Li Z. Yap is essential for treg-mediated suppression of antitumor immunity. Cancer Discovery. (2018) 8:1026–43. doi: 10.1158/2159-8290.Cd-17-1124 PMC6481611 29907586

[71] Myers JA Miller JS. Exploring the nk cell platform for cancer immunotherapy. Nat Rev Clin Oncol. (2021) 18:85–100. doi: 10.1038/s41571-020-0426-7 32934330 PMC8316981

[72] Morvan MG Lanier LL. Nk cells and cancer: you can teach innate cells new tricks. Nat Rev Cancer. (2016) 16:7–19. doi: 10.1038/nrc.2015.5 26694935

[73] Guillerey C Huntington ND Smyth MJ. Targeting natural killer cells in cancer immunotherapy. Nat Immunol. (2016) 17:1025–36. doi: 10.1038/ni.3518 27540992

[74] Feng S Sun H Zhu W. Mir-92 overexpression suppresses immune cell function in ovarian cancer *via* lats2/yap1/pd-L1 pathway. Clin Transl Oncol. (2021) 23:450–8. doi: 10.1007/s12094-020-02439-y 32654106

[75] Zhang H Zhu Y Wang J Weng S Zuo F Li C. Pkcι Regulates the expression of pdl1 through multiple pathways to modulate immune suppression of pancreatic cancer cells. Cell Signal. (2021) 86:110115. doi: 10.1016/j.cellsig.2021.110115 34375670

[76] Adhikary G Heipertz EL Preradovic M Chen X Xu W Newland JJ. Natural killer cells suppress human cutaneous squamous cell carcinoma cancer cell survival and tumor growth. Mol Carcinog. (2023) 62:845–54. doi: 10.1002/mc.23528 36994661

[77] Xu X Wang B Liu Y Jing T Xu G Zhang L. Etv4 potentiates nuclear yap retention and activities to enhance the progression of hepatocellular carcinoma. Cancer Lett. (2022) 537:215640. doi: 10.1016/j.canlet.2022.215640 35296440

[78] Xie M Lin Z Ji X Luo X Zhang Z Sun M. Fgf19/fgfr4-mediated elevation of etv4 facilitates hepatocellular carcinoma metastasis by upregulating pd-L1 and ccl2. J Hepatol. (2023) 79:109–25. doi: 10.1016/j.jhep.2023.02.036 36907560

[79] Zhang X Fu X Li T Yan H. The prognostic value of myeloid derived suppressor cell level in hepatocellular carcinoma: A systematic review and meta-analysis. PloS One. (2019) 14:e0225327. doi: 10.1371/journal.pone.0225327 31790437 PMC6886785

[80] Prieto J Melero I Sangro B. Immunological landscape and immunotherapy of hepatocellular carcinoma. Nat Rev Gastroenterol Hepatol. (2015) 12:681–700. doi: 10.1038/nrgastro.2015.173 26484443

[81] Li H Han Y Guo Q Zhang M Cao X. Cancer-expanded myeloid-derived suppressor cells induce anergy of nk cells through membrane-bound tgf-beta 1. J Immunol. (2009) 182:240–9. doi: 10.4049/jimmunol.182.1.240 19109155

[82] Murakami S Shahbazian D Surana R Zhang W Chen H Graham GT. Yes-associated protein mediates immune reprogramming in pancreatic ductal adenocarcinoma. Oncogene. (2017) 36:1232–44. doi: 10.1038/onc.2016.288 PMC5322249 27546622

[83] An Y Adams JR Hollern DP Zhao A Chang SG Gams MS. Cdh1 and pik3ca mutations cooperate to induce immune-related invasive lobular carcinoma of the breast. Cell Rep. (2018) 25:702–14.e6. doi: 10.1016/j.celrep.2018.09.056 30332649 PMC6276789

[84] Shibata M Ham K Hoque MO. A time for yap1: tumorigenesis, immunosuppression and targeted therapy. Int J Cancer. (2018) 143:2133–44. doi: 10.1002/ijc.31561 PMC6540999 29696628

[85] Hu X Zhang Y Yu H Zhao Y Sun X Li Q. The role of yap1 in survival prediction, immune modulation, and drug response: A pan-cancer perspective. Front Immunol. (2022) 13:1012173. doi: 10.3389/fimmu.2022.1012173 36479120 PMC9719955

[86] Affo S Yu LX Schwabe RF. The role of cancer-associated fibroblasts and fibrosis in liver cancer. Annu Rev Pathol. (2017) 12:153–86. doi: 10.1146/annurev-pathol-052016-100322 PMC5720358 27959632

[87] Akkız H. Emerging role of cancer-associated fibroblasts in progression and treatment of hepatocellular carcinoma. Int J Mol Sci. (2023) 24(4):3941. doi: 10.3390/ijms24043941 36835352 PMC9964606

[88] Mazzocca A Fransvea E Dituri F Lupo L Antonaci S Giannelli G. Down-regulation of connective tissue growth factor by inhibition of transforming growth factor beta blocks the tumor-stroma cross-talk and tumor progression in hepatocellular carcinoma. Hepatology. (2010) 51:523–34. doi: 10.1002/hep.23285 19821534

[89] Yamamura Y Asai N Enomoto A Kato T Mii S Kondo Y. Akt-girdin signaling in cancer-associated fibroblasts contributes to tumor progression. Cancer Res. (2015) 75:813–23. doi: 10.1158/0008-5472.Can-14-1317 25732845

[90] Mao X Xu J Wang W Liang C Hua J Liu J. Crosstalk between cancer-associated fibroblasts and immune cells in the tumor microenvironment: new findings and future perspectives. Mol Cancer. (2021) 20:131. doi: 10.1186/s12943-021-01428-1 34635121 PMC8504100

[91] Kubo N Araki K Kuwano H Shirabe K. Cancer-associated fibroblasts in hepatocellular carcinoma. World J Gastroenterol. (2016) 22:6841–50. doi: 10.3748/wjg.v22.i30.6841 PMC4974583 27570421

[92] Giannelli G Villa E Lahn M. Transforming growth factor-B as a therapeutic target in hepatocellular carcinoma. Cancer Res. (2014) 74:1890–4. doi: 10.1158/0008-5472.Can-14-0243 24638984

[93] Zhao H Yang L Baddour J Achreja A Bernard V Moss T. Tumor microenvironment derived exosomes pleiotropically modulate cancer cell metabolism. Elife. (2016) 5:e10250. doi: 10.7554/eLife.10250 26920219 PMC4841778

[94] Dupont S Morsut L Aragona M Enzo E Giulitti S Cordenonsi M. Role of yap/taz in mechanotransduction. Nature. (2011) 474:179–83. doi: 10.1038/nature10137 21654799

[95] Choi HJ Zhang H Park H Choi KS Lee HW Agrawal V. Yes-associated protein regulates endothelial cell contact-mediated expression of angiopoietin-2. Nat Commun. (2015) 6:6943. doi: 10.1038/ncomms7943 25962877

[96] Maller O DuFort CC Weaver VM. Yap forces fibroblasts to feel the tension. Nat Cell Biol. (2013) 15:570–2. doi: 10.1038/ncb2777 23728464

[97] Lee PJ Sui YH Liu TT Tsang NM Huang CH Lin TY. Epstein-barr viral product-containing exosomes promote fibrosis and nasopharyngeal carcinoma progression through activation of yap1/fapα Signaling in fibroblasts. J Exp Clin Cancer Res. (2022) 41:254. doi: 10.1186/s13046-022-02456-5 35986369 PMC9392321

[98] Huang R Liu J Li H Zheng L Jin H Zhang Y. Identification of hub genes and their correlation with immune infiltration cells in hepatocellular carcinoma based on geo and tcga databases. Front Genet. (2021) 12:647353. doi: 10.3389/fgene.2021.647353 33995482 PMC8120231

[99] Grivennikov SI Greten FR Karin M. Immunity, inflammation, and cancer. Cell. (2010) 140:883–99. doi: 10.1016/j.cell.2010.01.025 PMC2866629 20303878

[100] Zhang Q He Y Luo N Patel SJ Han Y Gao R. Landscape and dynamics of single immune cells in hepatocellular carcinoma. Cell. (2019) 179:829–45.e20. doi: 10.1016/j.cell.2019.10.003 31675496

[101] Wan S Zhao E Kryczek I Vatan L Sadovskaya A Ludema G. Tumor-associated macrophages produce interleukin 6 and signal *via* stat3 to promote expansion of human hepatocellular carcinoma stem cells. Gastroenterology. (2014) 147:1393–404. doi: 10.1053/j.gastro.2014.08.039 PMC4253315 25181692

[102] Zhu XD Zhang JB Zhuang PY Zhu HG Zhang W Xiong YQ. High expression of macrophage colony-stimulating factor in peritumoral liver tissue is associated with poor survival after curative resection of hepatocellular carcinoma. J Clin Oncol. (2008) 26:2707–16. doi: 10.1200/jco.2007.15.6521 18509183

[103] Zhou TY Zhou YL Qian MJ Fang YZ Ye S Xin WX. Interleukin-6 induced by yap in hepatocellular carcinoma cells recruits tumor-associated macrophages. J Pharmacol Sci. (2018) 138:89–95. doi: 10.1016/j.jphs.2018.07.013 30340922

[104] Naugler WE Sakurai T Kim S Maeda S Kim K Elsharkawy AM. Gender disparity in liver cancer due to sex differences in myd88-dependent il-6 production. Science. (2007) 317:121–4. doi: 10.1126/science.1140485 17615358

[105] Grivennikov S Karin E Terzic J Mucida D Yu GY Vallabhapurapu S. Il-6 and stat3 are required for survival of intestinal epithelial cells and development of colitis-associated cancer. Cancer Cell. (2009) 15:103–13. doi: 10.1016/j.ccr.2009.01.001 PMC2667107 19185845

[106] Roca H Varsos ZS Sud S Craig MJ Ying C Pienta KJ. Ccl2 and interleukin-6 promote survival of human cd11b+ Peripheral blood mononuclear cells and induce M2-type macrophage polarization. J Biol Chem. (2009) 284:34342–54. doi: 10.1074/jbc.M109.042671 PMC2797202 19833726

[107] Chen Y Song Y Du W Gong L Chang H Zou Z. Tumor-associated macrophages: an accomplice in solid tumor progression. J BioMed Sci. (2019) 26:78. doi: 10.1186/s12929-019-0568-z 31629410 PMC6800990

[108] Kim W Khan SK Liu Y Xu R Park O He Y. Hepatic hippo signaling inhibits protumoural microenvironment to suppress hepatocellular carcinoma. Gut. (2018) 67:1692–703. doi: 10.1136/gutjnl-2017-314061 PMC6592016 28866620

[109] Ning Z Guo X Liu X Lu C Wang A Wang X. Usp22 regulates lipidome accumulation by stabilizing pparγ in hepatocellular carcinoma. Nat Commun. (2022) 13:2187. doi: 10.1038/s41467-022-29846-9 35449157 PMC9023467

[110] Yang F Hilakivi-Clarke L Shaha A Wang Y Wang X Deng Y. Metabolic reprogramming and its clinical implication for liver cancer. Hepatology. (2023) 78:1602–24. doi: 10.1097/hep.0000000000000005 PMC10315435 36626639

[111] Du D Liu C Qin M Zhang X Xi T Yuan S. Metabolic dysregulation and emerging therapeutical targets for hepatocellular carcinoma. Acta Pharm Sin B. (2022) 12:558–80. doi: 10.1016/j.apsb.2021.09.019 PMC8897153 35256934

[112] Koo JH Guan KL. Interplay between yap/taz and metabolism. Cell Metab. (2018) 28:196–206. doi: 10.1016/j.cmet.2018.07.010 30089241

[113] Ganapathy-Kanniappan S Geschwind JF. Tumor glycolysis as a target for cancer therapy: progress and prospects. Mol Cancer. (2013) 12:152. doi: 10.1186/1476-4598-12-152 24298908 PMC4223729

[114] Feng J Li J Wu L Yu Q Ji J Wu J. Emerging roles and the regulation of aerobic glycolysis in hepatocellular carcinoma. J Exp Clin Cancer Res. (2020) 39:126. doi: 10.1186/s13046-020-01629-4 32631382 PMC7336654

[115] Ganapathy-Kanniappan S. Linking tumor glycolysis and immune evasion in cancer: emerging concepts and therapeutic opportunities. Biochim Biophys Acta Rev Cancer. (2017) 1868:212–20. doi: 10.1016/j.bbcan.2017.04.002 28400131

[116] Xu S Herschman HR. A tumor agnostic therapeutic strategy for hexokinase 1-null/hexokinase 2-positive cancers. Cancer Res. (2019) 79:5907–14. doi: 10.1158/0008-5472.Can-19-1789 PMC12139393 31434645

[117] Feng J Wu L Ji J Chen K Yu Q Zhang J. Pkm2 is the target of proanthocyanidin B2 during the inhibition of hepatocellular carcinoma. J Exp Clin Cancer Res. (2019) 38:204. doi: 10.1186/s13046-019-1194-z 31101057 PMC6525465

[118] Gatenby RA Gillies RJ. Why do cancers have high aerobic glycolysis? Nat Rev Cancer. (2004) 4:891–9. doi: 10.1038/nrc1478 15516961

[119] Feng J Dai W Mao Y Wu L Li J Chen K. Simvastatin re-sensitizes hepatocellular carcinoma cells to sorafenib by inhibiting hif-1α/ppar-Γ/pkm2-mediated glycolysis. J Exp Clin Cancer Res. (2020) 39:24. doi: 10.1186/s13046-020-1528-x 32000827 PMC6993409

[120] Wang L Yang Q Peng S Liu X. The combination of the glycolysis inhibitor 2-dg and sorafenib can be effective against sorafenib-tolerant persister cancer cells. Onco Targets Ther. (2019) 12:5359–73. doi: 10.2147/ott.S212465 PMC6635829 31371980

[121] Ward PS Thompson CB. Metabolic reprogramming: A cancer hallmark even Warburg did not anticipate. Cancer Cell. (2012) 21:297–308. doi: 10.1016/j.ccr.2012.02.014 22439925 PMC3311998

[122] Jang M Kim SS Lee J. Cancer cell metabolism: implications for therapeutic targets. Exp Mol Med. (2013) 45:e45. doi: 10.1038/emm.2013.85 24091747 PMC3809361

[123] Li J Zhu C Yue P Zheng T Li Y Wang B. Identification of glycolysis related pathways in pancreatic adenocarcinoma and liver hepatocellular carcinoma based on tcga and geo datasets. Cancer Cell Int. (2021) 21:128. doi: 10.1186/s12935-021-01809-y 33607990 PMC7893943

[124] Zhang X Li Y Ma Y Yang L Wang T Meng X. Yes-associated protein (Yap) binds to hif-1α and sustains hif-1α Protein stability to promote hepatocellular carcinoma cell glycolysis under hypoxic stress. J Exp Clin Cancer Res. (2018) 37:216. doi: 10.1186/s13046-018-0892-2 30180863 PMC6123950

[125] Chen R Zhu S Fan XG Wang H Lotze MT Zeh HJ 3rd. High mobility group protein B1 controls liver cancer initiation through yes-associated protein -dependent aerobic glycolysis. Hepatology. (2018) 67:1823–41. doi: 10.1002/hep.29663 PMC5906197 29149457

[126] Peng Q Hao L Guo Y Zhang Z Ji J Xue Y. Dihydroartemisinin inhibited the warburg effect through yap1/slc2a1 pathway in hepatocellular carcinoma. J Nat Med. (2023) 77:28–40. doi: 10.1007/s11418-022-01641-2 36068393

[127] Santinon G Pocaterra A Dupont S. Control of yap/taz activity by metabolic and nutrient-sensing pathways. Trends Cell Biol. (2016) 26:289–99. doi: 10.1016/j.tcb.2015.11.004 26750334

[128] Enzo E Santinon G Pocaterra A Aragona M Bresolin S Forcato M. Aerobic glycolysis tunes yap/taz transcriptional activity. EMBO J. (2015) 34:1349–70. doi: 10.15252/embj.201490379 PMC4491996 25796446

[129] Litwack G. Chapter 8 - glycolysis and gluconeogenesis. In: Litwack G, editor. Human Biochemistry, 2nd ed. Academic Press, Boston (2022). p. 207–25.

[130] Wang Z Dong C. Gluconeogenesis in cancer: function and regulation of pepck, fbpase, and G6pase. Trends Cancer. (2019) 5:30–45. doi: 10.1016/j.trecan.2018.11.003 30616754

[131] Grasmann G Smolle E Olschewski H Leithner K. Gluconeogenesis in cancer cells - repurposing of a starvation-induced metabolic pathway? Biochim Biophys Acta Rev Cancer. (2019) 1872:24–36. doi: 10.1016/j.bbcan.2019.05.006 31152822 PMC6894939

[132] Pavlova NN Thompson CB. The emerging hallmarks of cancer metabolism. Cell Metab. (2016) 23:27–47. doi: 10.1016/j.cmet.2015.12.006 26771115 PMC4715268

[133] Hirata H Sugimachi K Komatsu H Ueda M Masuda T Uchi R. Decreased expression of fructose-1,6-bisphosphatase associates with glucose metabolism and tumor progression in hepatocellular carcinoma. Cancer Res. (2016) 76:3265–76. doi: 10.1158/0008-5472.Can-15-2601 27197151

[134] Leithner K. Epigenetic marks repressing gluconeogenesis in liver and kidney cancer. Cancer Res. (2020) 80:657–8. doi: 10.1158/0008-5472.Can-19-3953 32060227

[135] Xiang J Chen C Liu R Gou D Chang L Deng H. Gluconeogenic enzyme pck1 deficiency promotes chk2 O-glcnacylation and hepatocellular carcinoma growth upon glucose deprivation. J Clin Invest. (2021) 131(8):e144703. doi: 10.1172/jci144703 33690219 PMC8262473

[136] Hu Y Shin DJ Pan H Lin Z Dreyfuss JM Camargo FD. Yap suppresses gluconeogenic gene expression through pgc1α. Hepatology. (2017) 66:2029–41. doi: 10.1002/hep.29373 PMC6082140 28714135

[137] Martin-Perez M Urdiroz-Urricelqui U Bigas C Benitah SA. The role of lipids in cancer progression and metastasis. Cell Metab. (2022) 34:1675–99. doi: 10.1016/j.cmet.2022.09.023 36261043

[138] Broadfield LA Pane AA Talebi A Swinnen JV Fendt SM. Lipid metabolism in cancer: new perspectives and emerging mechanisms. Dev Cell. (2021) 56:1363–93. doi: 10.1016/j.devcel.2021.04.013 33945792

[139] Snaebjornsson MT Janaki-Raman S Schulze A. Greasing the wheels of the cancer machine: the role of lipid metabolism in cancer. Cell Metab. (2020) 31:62–76. doi: 10.1016/j.cmet.2019.11.010 31813823

[140] Ye Y Sun X Lu Y. Obesity-related fatty acid and cholesterol metabolism in cancer-associated host cells. Front Cell Dev Biol. (2020) 8:600350. doi: 10.3389/fcell.2020.600350 33330490 PMC7729017

[141] Caro P Kishan AU Norberg E Stanley IA Chapuy B Ficarro SB. Metabolic signatures uncover distinct targets in molecular subsets of diffuse large B cell lymphoma. Cancer Cell. (2012) 22:547–60. doi: 10.1016/j.ccr.2012.08.014 PMC3479446 23079663

[142] Currie E Schulze A Zechner R Walther TC Farese RV Jr. Cellular fatty acid metabolism and cancer. Cell Metab. (2013) 18:153–61. doi: 10.1016/j.cmet.2013.05.017 PMC3742569 23791484

[143] Pope ED 3rd Kimbrough EO Vemireddy LP Surapaneni PK Copland JA 3rd Mody K. Aberrant lipid metabolism as a therapeutic target in liver cancer. Expert Opin Ther Targets. (2019) 23:473–83. doi: 10.1080/14728222.2019.1615883 PMC6594827 31076001

[144] Gu L Zhu Y Lin X Tan X Lu B Li Y. Stabilization of fasn by acat1-mediated gnpat acetylation promotes lipid metabolism and hepatocarcinogenesis. Oncogene. (2020) 39:2437–49. doi: 10.1038/s41388-020-1156-0 31974474

[145] Che L Chi W Qiao Y Zhang J Song X Liu Y. Cholesterol biosynthesis supports the growth of hepatocarcinoma lesions depleted of fatty acid synthase in mice and humans. Gut. (2020) 69:177–86. doi: 10.1136/gutjnl-2018-317581 PMC6943247 30954949

[146] Cai J Chen T Jiang Z Yan J Ye Z Ruan Y. Bulk and single-cell transcriptome profiling reveal extracellular matrix mechanical regulation of lipid metabolism reprograming through yap/tead4/acadl axis in hepatocellular carcinoma. Int J Biol Sci. (2023) 19:2114–31. doi: 10.7150/ijbs.82177 PMC10158031 37151879

[147] Gao Y Gong Y Liu Y Xue Y Zheng K Guo Y. Integrated analysis of transcriptomics and metabolomics in human hepatocellular carcinoma hepg2215 cells after yap1 knockdown. Acta Histochem. (2023) 125:151987. doi: 10.1016/j.acthis.2022.151987 36473310

[148] Cruz ALS Carrossini N Teixeira LK Ribeiro-Pinto LF Bozza PT Viola JPB. Cell cycle progression regulates biogenesis and cellular localization of lipid droplets. Mol Cell Biol. (2019) 39(9):e00374-18. doi: 10.1128/mcb.00374-18 30782775 PMC6469922

[149] Chi Y Gong Z Xin H Wang Z Liu Z. Long noncoding rna lncarsr promotes nonalcoholic fatty liver disease and hepatocellular carcinoma by promoting yap1 and activating the irs2/akt pathway. J Transl Med. (2020) 18:126. doi: 10.1186/s12967-020-02225-y 32169080 PMC7071718

[150] Lee CK Jeong SH Jang C Bae H Kim YH Park I. Tumor metastasis to lymph nodes requires yap-dependent metabolic adaptation. Science. (2019) 363:644–9. doi: 10.1126/science.aav0173 30733421

[151] Yan YC Meng GX Yang CC Yang YF Tan SY Yan LJ. Diacylglycerol lipase alpha promotes hepatocellular carcinoma progression and induces lenvatinib resistance by enhancing yap activity. Cell Death Dis. (2023) 14:404. doi: 10.1038/s41419-023-05919-5 37414748 PMC10325985

[152] Zhou TY Zhuang LH Hu Y Zhou YL Lin WK Wang DD. Inactivation of hypoxia-induced yap by statins overcomes hypoxic resistance tosorafenib in hepatocellular carcinoma cells. Sci Rep. (2016) 6:30483. doi: 10.1038/srep30483 27476430 PMC4967870

[153] Dessen A Tang J Schmidt H Stahl M Clark JD Seehra J. Crystal structure of human cytosolic phospholipase A2 reveals a novel topology and catalytic mechanism. Cell. (1999) 97:349–60. doi: 10.1016/s0092-8674(00)80744-8 10319815

[154] Yarla NS Bishayee A Sethi G Reddanna P Kalle AM Dhananjaya BL. Targeting arachidonic acid pathway by natural products for cancer prevention and therapy. Semin Cancer Biol. (2016) 40-41:48–81. doi: 10.1016/j.semcancer.2016.02.001 26853158

[155] Xu YJ Zheng Z Cao C Li J Liu Y. Bioanalytical insights into the association between eicosanoids and pathogenesis of hepatocellular carcinoma. Cancer Metastasis Rev. (2018) 37:269–77. doi: 10.1007/s10555-018-9747-8 29934821

[156] Chiu AP Tschida BR Sham TT Lo LH Moriarity BS Li XX. Hbx-K130m/V131i promotes liver cancer in transgenic mice *via* akt/foxo1 signaling pathway and arachidonic acid metabolism. Mol Cancer Res. (2019) 17:1582–93. doi: 10.1158/1541-7786.Mcr-18-1127 PMC6610579 30975706

[157] Wang K Shi JH Gao J Sun Y Wang Z Shi X. Arachidonic acid metabolism cyp450 pathway is deregulated in hepatocellular carcinoma and associated with microvascular invasion. Cell Biol Int. (2023) 48(1):31–45. doi: 10.1002/cbin.12086 37655528

[158] Liu Z Wang J Liu L Yuan H Bu Y Feng J. Chronic ethanol consumption and hbv induce abnormal lipid metabolism through hbx/swell1/arachidonic acid signaling and activate tregs in hbv-tg mice. Theranostics. (2020) 10:9249–67. doi: 10.7150/thno.46005 PMC7415795 32802190

[159] Sun L Suo C Zhang T Shen S Gu X Qiu S. Eno1 promotes liver carcinogenesis through yap1-dependent arachidonic acid metabolism. Nat Chem Biol. (2023) 19:1492–503. doi: 10.1038/s41589-023-01391-6 37500770

[160] Lieu EL Nguyen T Rhyne S Kim J. Amino acids in cancer. Exp Mol Med. (2020) 52:15–30. doi: 10.1038/s12276-020-0375-3 31980738 PMC7000687

[161] Green CR Wallace M Divakaruni AS Phillips SA Murphy AN Ciaraldi TP. Branched-chain amino acid catabolism fuels adipocyte differentiation and lipogenesis. Nat Chem Biol. (2016) 12:15–21. doi: 10.1038/nchembio.1961 26571352 PMC4684771

[162] Son SM Park SJ Lee H Siddiqi F Lee JE Menzies FM. Leucine signals to mtorc1 *via* its metabolite acetyl-coenzyme A. Cell Metab. (2019) 29:192–201.e7. doi: 10.1016/j.cmet.2018.08.013 30197302 PMC6331339

[163] Choudhari SK Chaudhary M Bagde S Gadbail AR Joshi V. Nitric oxide and cancer: A review. World J Surg Oncol. (2013) 11:118. doi: 10.1186/1477-7819-11-118 23718886 PMC3669621

[164] Hensley CT Wasti AT DeBerardinis RJ. Glutamine and cancer: cell biology, physiology, and clinical opportunities. J Clin Invest. (2013) 123:3678–84. doi: 10.1172/jci69600 PMC3754270 23999442

[165] Bertero T Oldham WM Grasset EM Bourget I Boulter E Pisano S. Tumor-stroma mechanics coordinate amino acid availability to sustain tumor growth and Malignancy. Cell Metab. (2019) 29:124–40.e10. doi: 10.1016/j.cmet.2018.09.012 30293773 PMC6432652

[166] Hoadley KA Yau C Wolf DM Cherniack AD Tamborero D Ng S. Multiplatform Analysis of 12 Cancer Types Reveals Molecular Classification within and across Tissues of Origin. Cell. (2014) 158:929–44. doi: 10.1016/j.cell.2014.06.049 PMC4152462 25109877

[167] Yuneva MO Fan TW Allen TD Higashi RM Ferraris DV Tsukamoto T. The metabolic profile of tumors depends on both the responsible genetic lesion and tissue type. Cell Metab. (2012) 15:157–70. doi: 10.1016/j.cmet.2011.12.015 PMC3282107 22326218

[168] Nilsson A Haanstra JR Engqvist M Gerding A Bakker BM Klingmüller U. Quantitative analysis of amino acid metabolism in liver cancer links glutamate excretion to nucleotide synthesis. Proc Natl Acad Sci U.S.A. (2020) 117:10294–304. doi: 10.1073/pnas.1919250117 PMC7229649 32341162

[169] Li Y Mo H Jia S Wang J Ma Y Liu X. Comprehensive analysis of the amino acid metabolism-related gene signature for prognosis, tumor immune microenvironment, and candidate drugs in hepatocellular carcinoma. Front Immunol. (2022) 13:1066773. doi: 10.3389/fimmu.2022.1066773 36582227 PMC9792509

[170] Yang D Liu H Cai Y Lu K Zhong X Xing S. Branched-chain amino acid catabolism breaks glutamine addiction to sustain hepatocellular carcinoma progression. Cell Rep. (2022) 41:111691. doi: 10.1016/j.celrep.2022.111691 36417878

[171] Namikawa M Kakizaki S Kaira K Tojima H Yamazaki Y Horiguchi N. Expression of amino acid transporters (Lat1, asct2 and xct) as clinical significance in hepatocellular carcinoma. Hepatol Res. (2015) 45:1014–22. doi: 10.1111/hepr.12431 25297701

[172] Saito Y Soga T. Amino acid transporters as emerging therapeutic targets in cancer. Cancer Sci. (2021) 112:2958–65. doi: 10.1111/cas.15006 PMC8353895 34091991

[173] Cox AG Hwang KL Brown KK Evason K Beltz S Tsomides A. Yap reprograms glutamine metabolism to increase nucleotide biosynthesis and enable liver growth. Nat Cell Biol. (2016) 18:886–96. doi: 10.1038/ncb3389 PMC4990146 27428308

[174] Hansen CG Ng YL Lam WL Plouffe SW Guan KL. The hippo pathway effectors yap and taz promote cell growth by modulating amino acid signaling to mtorc1. Cell Res. (2015) 25:1299–313. doi: 10.1038/cr.2015.140 PMC4670996 26611634

[175] Lu X Peng B Chen G Pes MG Ribback S Ament C. Yap accelerates notch-driven cholangiocarcinogenesis *via* mtorc1 in mice. Am J Pathol. (2021) 191:1651–67. doi: 10.1016/j.ajpath.2021.05.017 PMC8420864 34129844

[176] Hanahan D Weinberg RA. Hallmarks of cancer: the next generation. Cell. (2011) 144:646–74. doi: 10.1016/j.cell.2011.02.013 21376230

[177] Cheng Y Li H Deng Y Tai Y Zeng K Zhang Y. Cancer-associated fibroblasts induce pdl1+ Neutrophils through the il6-stat3 pathway that foster immune suppression in hepatocellular carcinoma. Cell Death Dis. (2018) 9:422. doi: 10.1038/s41419-018-0458-4 29556041 PMC5859264

[178] Perra A Kowalik MA Ghiso E Ledda-Columbano GM Di Tommaso L Angioni MM. Yap activation is an early event and a potential therapeutic target in liver cancer development. J Hepatol. (2014) 61:1088–96. doi: 10.1016/j.jhep.2014.06.033 25010260

[179] Yu M Peng Z Qin M Liu Y Wang J Zhang C. Interferon-Γ Induces tumor resistance to anti-pd-1 immunotherapy by promoting yap phase separation. Mol Cell. (2021) 81:1216–30.e9. doi: 10.1016/j.molcel.2021.01.010 33606996

[180] Zhuang Y Wang Y Liu C Li S Du S Li G. Yes-associated protein 1 inhibition induces immunogenic cell death and synergizes with radiation and pd-1 blockade. Int J Radiat Oncol Biol Phys. (2023) 116:894–905. doi: 10.1016/j.ijrobp.2022.12.045 36608830

[181] Guo Y Peng Q Hao L Ji J Zhang Z Xue Y. Dihydroartemisinin promoted fxr expression independent of yap1 in hepatocellular carcinoma. FASEB J. (2022) 36:e22361. doi: 10.1096/fj.202200171R 35616366

[182] Zhao S Xu K Jiang R Li DY Guo XX Zhou P. Evodiamine inhibits proliferation and promotes apoptosis of hepatocellular carcinoma cells *via* the hippo-yes-associated protein signaling pathway. Life Sci. (2020) 251:117424. doi: 10.1016/j.lfs.2020.117424 32057900

[183] Yun UJ Bae SJ Song YR Kim YW. A critical yap in Malignancy of hcc is regulated by evodiamine. Int J Mol Sci. (2022) 23(3):1855. doi: 10.3390/ijms23031855 35163776 PMC8837083

[184] Li J Wang H Wang L Tan R Zhu M Zhong X. Decursin inhibits the growth of hepg2 hepatocellular carcinoma cells *via* hippo/yap signaling pathway. Phytother Res. (2018) 32:2456–65. doi: 10.1002/ptr.6184 30251417

[185] Wang L Zhu Z Han L Zhao L Weng J Yang H. A curcumin derivative, wz35, suppresses hepatocellular cancer cell growth *via* downregulating yap-mediated autophagy. Food Funct. (2019) 10:3748–57. doi: 10.1039/c8fo02448k 31172987

[186] Wang L Zhu Z Liang Q Tao Y Jin G Zhong Y. A novel small molecule glycolysis inhibitor wz35 exerts anti-cancer effect *via* metabolic reprogramming. J Transl Med. (2022) 20:530. doi: 10.1186/s12967-022-03758-0 36401321 PMC9673307

[187] Wu C Kan H Hu M Liu X Boye A Jiang Y. Compound astragalus and salvia miltiorrhiza extract inhibits hepatocarcinogenesis *via* modulating tgf-B/Tβr and imp7/8. Exp Ther Med. (2018) 16:1052–60. doi: 10.3892/etm.2018.6292 PMC6090435 30112050

[188] Xu W Shi Z Yu X Xu Y Chen Y He Y. Salvianolic acid B exerts an anti-hepatocellular carcinoma effect by regulating the hippo/yap pathway and promoting psmad3l to psmad3c simultaneously. Eur J Pharmacol. (2023) 939:175423. doi: 10.1016/j.ejphar.2022.175423 36509132

[189] Xia H Dai X Yu H Zhou S Fan Z Wei G. Egfr-pi3k-pdk1 pathway regulates yap signaling in hepatocellular carcinoma: the mechanism and its implications in targeted therapy. Cell Death Dis. (2018) 9:269. doi: 10.1038/s41419-018-0302-x 29449645 PMC5833379

[190] Benhammou JN Qiao B Ko A Sinnett-Smith J Pisegna JR Rozengurt E. Lipophilic statins inhibit yap coactivator transcriptional activity in hcc cells through rho-mediated modulation of actin cytoskeleton. Am J Physiol Gastrointest Liver Physiol. (2023) 325:G239–g50. doi: 10.1152/ajpgi.00089.2023 PMC10511177 37366601

[191] Brown DM Kaiser PK Michels M Soubrane G Heier JS Kim RY. Ranibizumab versus verteporfin for neovascular age-related macular degeneration. N Engl J Med. (2006) 355:1432–44. doi: 10.1056/NEJMoa062655 17021319

[192] Song S Ajani JA Honjo S Maru DM Chen Q Scott AW. Hippo coactivator yap1 upregulates sox9 and endows esophageal cancer cells with stem-like properties. Cancer Res. (2014) 74:4170–82. doi: 10.1158/0008-5472.Can-13-3569 PMC4136429 24906622

[193] Gibault F Bailly F Corvaisier M Coevoet M Huet G Melnyk P. Molecular features of the yap inhibitor verteporfin: synthesis of hexasubstituted dipyrrins as potential inhibitors of yap/taz, the downstream effectors of the hippo pathway. ChemMedChem. (2017) 12:954–61. doi: 10.1002/cmdc.201700063 28334506

[194] Gong Y Peng Q Gao Y Yang J Lu J Zhang Y. Dihydroartemisinin inhibited interleukin-18 expression by decreasing yap1 in hepatocellular carcinoma cells. Acta Histochem. (2023) 125:152040. doi: 10.1016/j.acthis.2023.152040 37119608

[195] Gavini J Dommann N Jakob MO Keogh A Bouchez LC Karkampouna S. Verteporfin-induced lysosomal compartment dysregulation potentiates the effect of sorafenib in hepatocellular carcinoma. Cell Death Dis. (2019) 10:749. doi: 10.1038/s41419-019-1989-z 31582741 PMC6776510

[196] Sun T Mao W Peng H Wang Q Jiao L. Yap promotes sorafenib resistance in hepatocellular carcinoma by upregulating survivin. Cell Oncol (Dordr). (2021) 44:689–99. doi: 10.1007/s13402-021-00595-z PMC12980756 33655469

[197] Li X Ge J Zheng Q Zhang J Sun R Liu R. Evodiamine and rutaecarpine from tetradium ruticarpum in the treatment of liver diseases. Phytomedicine. (2020) 68:153180. doi: 10.1016/j.phymed.2020.153180 32092638

[198] Yu H Jin H Gong W Wang Z Liang H. Pharmacological actions of multi-target-directed evodiamine. Molecules. (2013) 18:1826–43. doi: 10.3390/molecules18021826 PMC6270287 23434865

[199] Yang S Chen J Tan T Wang N Huang Y Wang Y. Evodiamine Exerts Anticancer Effects against 143b and Mg63 Cells through the Wnt/B-Catenin Signaling Pathway. Cancer Manag Res. (2020) 12:2875–88. doi: 10.2147/cmar.S238093 PMC7196244 32425601

[200] Deng JD Lei S Jiang Y Zhang HH Hu XL Wen HX. A concise synthesis and biological study of evodiamine and its analogues. Chem Commun (Camb). (2019) 55:3089–92. doi: 10.1039/c9cc00434c 30785464

[201] Lin L Ren L Wen L Wang Y Qi J. Effect of evodiamine on the proliferation and apoptosis of A549 human lung cancer cells. Mol Med Rep. (2016) 14:2832–8. doi: 10.3892/mmr.2016.5575 27485202

[202] Hu C Gao X Han Y Guo Q Zhang K Liu M. Evodiamine Sensitizes Bgc-823 Gastric Cancer Cells to Radiotherapy In vitro and In vivo. Mol Med Rep. (2016) 14:413–9. doi: 10.3892/mmr.2016.5237 27176933

[203] Liu H Huang C Wu L Wen B. Effect of evodiamine and berberine on mir-429 as an oncogene in human colorectal cancer. Onco Targets Ther. (2016) 9:4121–7. doi: 10.2147/ott.S104729 PMC4940014 27462166

[204] Chen TC Chien CC Wu MS Chen YC. Evodiamine from evodia rutaecarpa induces apoptosis *via* activation of jnk and perk in human ovarian cancer cells. Phytomedicine. (2016) 23:68–78. doi: 10.1016/j.phymed.2015.12.003 26902409

[205] Hu CY Wu HT Su YC Lin CH Chang CJ Wu CL. Evodiamine exerts an anti-hepatocellular carcinoma activity through a wwox-dependent pathway. Molecules. (2017) 22(7):1175. doi: 10.3390/molecules22071175 28708106 PMC6152263

[206] Wen Z Feng S Wei L Wang Z Hong D Wang Q. Evodiamine, a novel inhibitor of the wnt pathway, inhibits the self-renewal of gastric cancer stem cells. Int J Mol Med. (2015) 36:1657–63. doi: 10.3892/ijmm.2015.2383 26497016

[207] Yang F Shi L Liang T Ji L Zhang G Shen Y. Anti-tumor effect of evodiamine by inducing akt-mediated apoptosis in hepatocellular carcinoma. Biochem Biophys Res Commun. (2017) 485:54–61. doi: 10.1016/j.bbrc.2017.02.017 28189683

[208] Shehzad A Parveen S Qureshi M Subhan F Lee YS. Decursin and decursinol angelate: molecular mechanism and therapeutic potential in inflammatory diseases. Inflammation Res. (2018) 67:209–18. doi: 10.1007/s00011-017-1114-7 29134229

[209] Choi H Yoon JH Youn K Jun M. Decursin prevents melanogenesis by suppressing mitf expression through the regulation of pka/creb, mapks, and pi3k/akt/gsk-3β Cascades. BioMed Pharmacother. (2022) 147:112651. doi: 10.1016/j.biopha.2022.112651 35063859

[210] Ge Y Yoon SH Jang H Jeong JH Lee YM. Decursin promotes hif-1α Proteasomal degradation and immune responses in hypoxic tumour microenvironment. Phytomedicine. (2020) 78:153318. doi: 10.1016/j.phymed.2020.153318 32896707

[211] Kim S Lee SI Kim N Joo M Lee KH Lee MW. Decursin inhibits cell growth and autophagic flux in gastric cancer *via* suppression of cathepsin C. Am J Cancer Res. (2021) 11:1304–20. PMC8085838 33948359

[212] Joo M Heo JB Kim S Kim N Jeon HJ An Y. Decursin inhibits tumor progression in head and neck squamous cell carcinoma by downregulating cxcr7 expression in vitro. Oncol Rep. (2022) 47(2):39. doi: 10.3892/or.2021.8250 34958113 PMC8759107

[213] Kim HJ Kim SM Park KR Jang HJ Na YS Ahn KS. Decursin chemosensitizes human multiple myeloma cells through inhibition of stat3 signaling pathway. Cancer Lett. (2011) 301:29–37. doi: 10.1016/j.canlet.2010.11.002 21122982

[214] Kim J Yun M Kim EO Jung DB Won G Kim B. Decursin enhances trail-induced apoptosis through oxidative stress mediated- endoplasmic reticulum stress signalling in non-small cell lung cancers. Br J Pharmacol. (2016) 173:1033–44. doi: 10.1111/bph.13408 PMC5341238 26661339

[215] Kim WJ Lee SJ Choi YD Moon SK. Decursin inhibits growth of human bladder and colon cancer cells *via* apoptosis, G1-phase cell cycle arrest and extracellular signal-regulated kinase activation. Int J Mol Med. (2010) 25:635–41. doi: 10.3892/ijmm_00000386 20198313

[216] Wang L Han L Tao Z Zhu Z Han L Yang Z. The curcumin derivative wz35 activates ros-dependent jnk to suppress hepatocellular carcinoma metastasis. Food Funct. (2018) 9:2970–8. doi: 10.1039/c8fo00314a 29766185

[217] Khan MA Zafaryab M Mehdi SH Ahmad I Rizvi MM. Characterization and anti-proliferative activity of curcumin loaded chitosan nanoparticles in cervical cancer. Int J Biol Macromol. (2016) 93:242–53. doi: 10.1016/j.ijbiomac.2016.08.050 27565296

[218] Gaikwad D Shewale R Patil V Mali D Gaikwad U Jadhav N. Enhancement in in vitro anti-angiogenesis activity and cytotoxicity in lung cancer cell by pectin-pvp based curcumin particulates. Int J Biol Macromol. (2017) 104:656–64. doi: 10.1016/j.ijbiomac.2017.05.170 28602990

[219] Chen T Chen J Zeng T Huang Q Chen D Chen H. Wz35 inhibits gastric cancer cell metastasis by depleting glutathione to promote cellular metabolic remodeling. Cancer Lett. (2023) 555:216044. doi: 10.1016/j.canlet.2022.216044 36574880

[220] He W Xia Y Cao P Hong L Zhang T Shen X. Curcuminoid wz35 synergize with cisplatin by inducing ros production and inhibiting trxr1 activity in gastric cancer cells. J Exp Clin Cancer Res. (2019) 38:207. doi: 10.1186/s13046-019-1215-y 31113439 PMC6528260

[221] Wang L Wang C Tao Z Zhao L Zhu Z Wu W. Curcumin derivative wz35 inhibits tumor cell growth *via* ros-yap-jnk signaling pathway in breast cancer. J Exp Clin Cancer Res. (2019) 38:460. doi: 10.1186/s13046-019-1424-4 31703744 PMC6842168

[222] Zhang J Feng Z Wang C Zhou H Liu W Kanchana K. Curcumin derivative wz35 efficiently suppresses colon cancer progression through inducing ros production and er stress-dependent apoptosis. Am J Cancer Res. (2017) 7:275–88. PMC5336501 28337376

[223] Zhang X Chen M Zou P Kanchana K Weng Q Chen W. Curcumin analog wz35 induced cell death *via* ros-dependent er stress and G2/M cell cycle arrest in human prostate cancer cells. BMC Cancer. (2015) 15:866. doi: 10.1186/s12885-015-1851-3 26546056 PMC4636884

[224] Dai X Zhang X Chen W Chen Y Zhang Q Mo S. Dihydroartemisinin: A potential natural anticancer drug. Int J Biol Sci. (2021) 17:603–22. doi: 10.7150/ijbs.50364 PMC7893584 33613116

[225] Klayman DL. Qinghaosu (Artemisinin): an antimalarial drug from China. Science. (1985) 228:1049–55. doi: 10.1126/science.3887571 3887571

[226] Renner K Bruss C Schnell A Koehl G Becker HM Fante M. Restricting glycolysis preserves T cell effector functions and augments checkpoint therapy. Cell Rep. (2019) 29:135–50.e9. doi: 10.1016/j.celrep.2019.08.068 31577944

[227] Gong Y Li D Li L Yang J Ding H Zhang C. Smad3 C-terminal phosphorylation site mutation attenuates the hepatoprotective effect of salvianolic acid B against hepatocarcinogenesis. Food Chem Toxicol. (2021) 147:111912. doi: 10.1016/j.fct.2020.111912 33290806

[228] Yang Y Pei K Zhang Q Wang D Feng H Du Z. Salvianolic acid B ameliorates atherosclerosis *via* inhibiting yap/taz/jnk signaling pathway in endothelial cells and pericytes. Biochim Biophys Acta Mol Cell Biol Lipids. (2020) 1865:158779. doi: 10.1016/j.bbalip.2020.158779 32739616

[229] Zhang N Hu Y Ding C Zeng W Shan W Fan H. Salvianolic acid B protects against chronic alcoholic liver injury *via* sirt1-mediated inhibition of crp and chrebp in rats. Toxicol Lett. (2017) 267:1–10. doi: 10.1016/j.toxlet.2016.12.010 27989594

[230] Pergolizzi JV Jr. Coluzzi F Colucci RD Olsson H LeQuang JA Al-Saadi J. Statins and muscle pain. Expert Rev Clin Pharmacol. (2020) 13:299–310. doi: 10.1080/17512433.2020.1734451 32089020

[231] Ward NC Watts GF Eckel RH. Statin toxicity. Circ Res. (2019) 124:328–50. doi: 10.1161/circresaha.118.312782 30653440

[232] Islam MM Poly TN Walther BA Yang HC Jack Li YC. Statin use and the risk of hepatocellular carcinoma: A meta-analysis of observational studies. Cancers (Basel). (2020) 12(3):671. doi: 10.3390/cancers12030671 32183029 PMC7139959

[233] Pinyopornpanish K Al-Yaman W Butler RS Carey W McCullough A Romero-Marrero C. Chemopreventive effect of statin on hepatocellular carcinoma in patients with nonalcoholic steatohepatitis cirrhosis. Am J Gastroenterol. (2021) 116:2258–69. doi: 10.14309/ajg.0000000000001347 34212895

[234] Simon TG Duberg AS Aleman S Hagstrom H Nguyen LH Khalili H. Lipophilic statins and risk for hepatocellular carcinoma and death in patients with chronic viral hepatitis: results from a nationwide swedish population. Ann Intern Med. (2019) 171:318–27. doi: 10.7326/m18-2753 PMC8246628 31426090

[235] Kim MH Kim MY Salloum S Qian T Wong LP Xu M. Atorvastatin favorably modulates a clinical hepatocellular carcinoma risk gene signature. Hepatol Commun. (2022) 6:2581–93. doi: 10.1002/hep4.1991 PMC9426409 35712812

[236] Wang T Rao D Yu C Sheng J Luo Y Xia L. Rho gtpase family in hepatocellular carcinoma. Exp Hematol Oncol. (2022) 11:91. doi: 10.1186/s40164-022-00344-4 36348464 PMC9644471

[237] Zhou W Liu H Yuan Z Zundell J Towers M Lin J. Targeting the mevalonate pathway suppresses arid1a-inactivated cancers by promoting pyroptosis. Cancer Cell. (2023) 41:740–56.e10. doi: 10.1016/j.ccell.2023.03.002 36963401 PMC10085864

